# PDK4-driven lactate accumulation facilitates LPCAT2 lactylation to exacerbate sepsis-induced acute lung injury

**DOI:** 10.1038/s41418-025-01585-6

**Published:** 2025-10-07

**Authors:** Yifan Deng, Yuetan Qiu, Xiang Li, Ting Gong, Jinyan Guo, Haoxuan Liang, Ziyi Yuan, Ziqing Hei, Xuedi Zhang, Youtan Liu

**Affiliations:** 1 Department of Anesthesiology, Shenzhen Hospital, Southern Medical University, Shenzhen, 518110 Guangdong China; 2 Shenzhen School of Clinical Medicine, Southern Medical University, Shenzhen, 518110 Guangdong China; 3 Department of Anesthesiology, The Third Affiliated Hospital of Sun Yat-Sen University, Guangzhou, 510630 Guangdong China; 4 Department of Anesthesiology, The First Affiliated Hospital of Guangxi Medical University, No. 6 Shuangyong Road, Qingxiu District, Nanning, 530021 Guangxi China

**Keywords:** Respiratory tract diseases, Protein-protein interaction networks

## Abstract

Elevated glycolysis in lung tissue is a hallmark of sepsis-induced acute lung injury (SI-ALI), yet the role of glycolytic reprogramming and lactate-derived protein modifications in damaging epithelial cells remains poorly understood. In this study, we reveal that PDK4-driven glycolytic reprogramming promotes excessive lactate production in lung tissue during SI-ALI. Mechanistically, AARS1 in epithelial cells selectively enhances lactylation modification at the K375 site of LPCAT2, which suppresses STAT1 acetylation and facilitates STAT1 phosphorylation, nuclear translocation, and transcriptional repression of SLC7A11. This cascade ultimately triggers epithelial cells ferroptosis. Pharmacological inhibition of PDK4 attenuates lactate accumulation and LPCAT2 lactylation, thereby restoring STAT1 acetylation and SLC7A11 expression. Furthermore, AARS1 knockdown or mutation of the LPCAT2-K375 lactylation site rescues STAT1-mediated SLC7A11 suppression and mitigates ferroptosis in vitro and septic mice. Our findings revealed that elevated expression of PDK4 is a critical factor contributing to the increased lactate production in lung tissue during sepsis, and established a novel LPCAT2-K375/STAT1/SLC7A11 axis driving epithelial cells ferroptosis in SI-ALI, highlighting the crosstalk between metabolic reprogramming, post-translational modifications (PTM), and ferroptosis. Targeting the PDK4 or LPCAT2 lactylation may offer therapeutic potential for SI-ALI.

**Figure:**
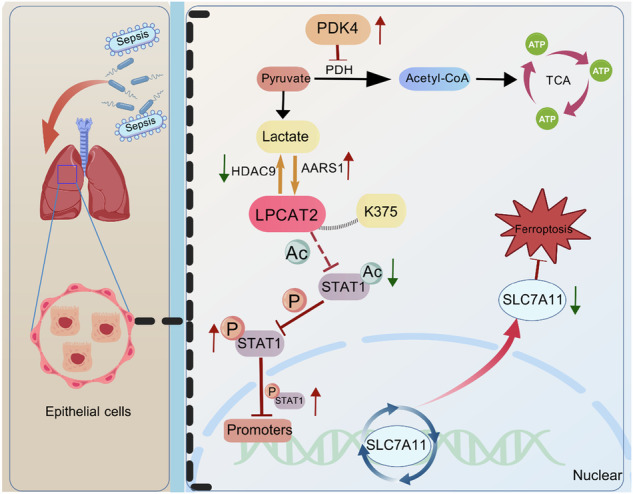
In sepsis-induced acute lung injury (SI-ALI), PDK4 hyperactivation drives excessive lactate production in epithelial cells, triggering AARS1/HDAC9-mediated LPCAT2 lactylation. This modification suppresses STAT1 acetylation while enhancing phosphorylation, driving its nuclear translocation and subsequent SLC7A11 transcriptional downregulation. The resultant glutathione synthesis deficiency promotes ferroptosis, exacerbating SI-ALI progression.

In sepsis-induced acute lung injury (SI-ALI), PDK4 hyperactivation drives excessive lactate production in epithelial cells, triggering AARS1/HDAC9-mediated LPCAT2 lactylation. This modification suppresses STAT1 acetylation while enhancing phosphorylation, driving its nuclear translocation and subsequent SLC7A11 transcriptional downregulation. The resultant glutathione synthesis deficiency promotes ferroptosis, exacerbating SI-ALI progression.

## Introduction

Sepsis-induced organ injury and multiple organ dysfunction syndrome remain the leading causes of mortality in critically ill patients [1, 2]. Among organs, the lung is considered the most vulnerable to injury and subsequent failure in septic patients [3]. Sepsis accounts for >50% of acute lung injury cases, with sepsis-induced acute lung injury (SI-ALI) progressing to fatal respiratory failure in 40% of untreated cases [4, 5]. This not only brings a devastating burden to patients and their families but also places a significant strain on healthcare resources. Therefore, elucidating the mechanisms underlying SI-ALI and developing effective prevention and treatment strategies are critical challenges in the field of critical care medicine, with profound social and economic implications.

Traditionally, lactate has been primarily regarded as a marker of tissue anaerobic metabolism [6]. In severe sepsis patients, lung lactate levels correlate positively with acute lung injury (ALI) severity and mortality [7, 8]. However, the molecular mechanisms underlying this increase remain poorly understood. Lung epithelial cells, acting as the primary barrier against inhaled pathogens, critically influence ALI progression. Studies confirm that epithelial denudation severity directly correlates with ALI intensity [9, 10], while barrier dysfunction is recognized as a central driver of multiorgan failure at the cellular level [11]. Considering lactate levels correlate with SI-ALI severity and lung epithelial cell injury, prioritizing research to clarify how lactate affects these cells could reveal critical mechanisms that aggravate SI-ALI.

Ferroptosis is an iron-dependent form of regulated cell death propelled by lipid peroxidation [12]. Previous evidence suggests that ferroptosis is progressively being regarded as a crucial driver in SI-ALI [13], while inhibiting ferroptosis alleviates SI-ALI [14]. Li et al. demonstrated that macrophage-derived extracellular vesicles carrying GBP2 promote ferroptosis in pulmonary vascular endothelial cells, thereby worsening SI-ALI [15]. Similarly, Sang et al. showed that METTL4-mediated N6-methyladenosine activates ferroptosis in alveolar epithelial cells, contributing to acute lung injury [3]. These findings collectively highlight the critical role of ferroptosis in SI-ALI. Lactate also significantly enhances the propensity for ferroptosis. For instance, lactate disrupts redox balance to increase ROS, damaging mitochondrial membranes and inducing ferroptosis [16]. It also promotes histone lactylation and METTL3-mediated m6A modification of ACSL4 to drive ferroptosis in SI-ALI [17]. While most studies focus on histone-regulated ferroptosis under high lactate, whether non-histone proteins regulate ferroptosis to exacerbate SI-ALI remains unaddressed.

Lactylation, a lactate-driven PTM [18], modifies histones/non-histone proteins via lysine lactylation [19, 20], regulating gene expression and metabolic reprogramming in SI-ALI. However, SI-ALI-specific lactylation sequencing data remain absent, limiting mechanistic understanding.

In the present study, mass spectrometry identified pyruvate dehydrogenase kinase 4 (PDK4) as driving glycolytic reprogramming and lactate accumulation in SI-ALI tissues. Lactylation sequencing identified lysophosphatidylcholine acyltransferase 2 (LPCAT2) as a significantly lactylated protein, which reduces acetylation of transcription activator 1 (STAT1) while enhancing its phosphorylation, suppressing solute carrier family 7 member 11 (SLC7A11) to trigger ferroptosis and exacerbate injury. Alanyl-tRNA synthetase-1 (AARS1) enhanced and histone deacetylase 9 (HDAC9) suppressed this modification. This study clarifies how PDK4-driven lactate exacerbates SI-ALI through LPCAT2 lactylation, advancing pathogenesis understanding and informing targeted therapeutic development.

## Materials and methods

### Ethnic statements

This study was approved by the Medical Ethics Committee of Shenzhen Hospital, Southern Medical University (No. SZYYEC2025K159R001). Healthy volunteers were recruited through hospital staff and public announcements. We prospectively enrolled adult patients (>18 years) admitted to the ICU between November 2018 and December 2021 who met the Sepsis-3 criteria within 24 h of admission. Written informed consent was obtained from all participants or their relatives. Patients were excluded if they were pregnant, immunocompromised, had leukopenia, hematologic malignancies, or were transitioning to palliative care. All animal procedures were approved by the Institutional Animal Care and Use Committee (IACUC) of the Laboratory Animal Center at South China Agricultural University (Approval No. 2024H045) and conducted in strict compliance with ARRIVE 2.0 guidelines, including humane endpoints and pain management protocols to ensure ethical rigor and reproducibility.

### Cecal ligation and puncture mouse model

The method of creating the Cecal ligation and puncture (CLP) model in C57BL/6 J mice was performed as before [21]. Briefly, isoflurane (induction concentration: 3–4%, maintenance concentration: 1.5–2.5%) was used for anesthesia. The abdominal wall was incised to expose the cecum, which was then ligated 15 mm from the apex with a 5-0 silk thread and perforated with a 21-gauge needle. After removing a bit of fecal content, the cecum was repositioned and the abdominal wall was sutured. All mice received 0.9% saline intraperitoneally for rehydration post-surgery. Some were sacrificed 24 h later for blood and lung tissue collection for research.

### In vivo treatment

8–10 weeks C57BL/6 J male mice were randomly divided into different groups (six mice per group). To explore the effect of lactate on SI-ALI, lactate (0.5 g/kg, Sigma-Aldrich, Saint Louis, USA, L6661) was injected intraperitoneally 6 h following CLP/sham surgery [22]. To suppress lactate production, hexokinase 2 (HK2) inhibitor 2-deoxy-D-glucose (2-DG, 0.5 g/kg, Selleck, Houston, USA, S4701) [23] or the PDK4 inhibitor PDK4-in (10, 20 or 30 mg/kg, MedChemExpress, Monmouth Junction, USA, 2310262-10-1) was administered intraperitoneally 3 h before CLP or sham operation to inhibit lactate production. In addition, UAMC-3203 (20 μmol/kg, MedChemExpress, HY-112909; administered via intravenous injection 15 min before CLP) [24] was used to explore the effect of ferroptosis on SI-ALI.

### Cell culture and treatments

BEAS-2B, an immortalized and non-tumorigenic human epithelial cell line that has been widely used to study lung-related cellular and molecular mechanisms [25], was obtained from the Beyotime Company (Shanghai, China). MLE12 cells, a mouse type II alveolar epithelial cell line sourced from the Cell Bank of the Chinese Academy of Sciences (Shanghai, China). All cells were authenticated by STR profiling and tested for mycoplasma contamination. BEAS-2B cells were maintained in Dulbecco’s Modified Eagle Medium (DMEM) supplemented with 10% fetal bovine serum (FBS; Biological Industries, Israel) and 1% penicillin-streptomycin solution at 37°C in a humidified atmosphere containing 5% CO2. To establish an in vitro model of lactate overload for investigating cellular metabolic dysregulation under hyperlactatemic conditions, BEAS-2B cells were stimulated with lactate (Lac, 10 mM, Beyotime, Shanghai, China, S0208S) for 24 h. To investigate the role of lactate in epithelial cell responses, BEAS-2B cells were co-treated with LPS (10 μg/ml, Beyotime, S1732) and the PDK4-IN (30, 40, or 50 μM) for 24 h. Fludarabine (10 μM, Selleck, S1229) [26], a specific inhibitor of STAT1 phosphorylation [26], was employed in the experimental design. In the cell viability experiments, the ferroptosis inhibitor Ferrostatin-1 (Fer-1, 2 μM, MedChemExpress, HY-100579) was also employed [27]. Other reagents, such as the PRMT1 inhibitor C7280948 (200 μM, Selleck Chemicals, Houston, USA, S6737) [28] and the ACSL4 inhibitor AS-252424 (10 μM, MedChemExpress, HY-13532), which was pre-treated with cells at a concentration of 10 μM for 1 h [29], were also employed in the study. Additionally, ATP (10 mM, Beyotime, D7378) was supplemented to facilitate the AARS1 enzymatic reaction in the in vitro cellular system [30]. To elucidate the role of lactate accumulation in inducing ferroptosis within the lung, MLE12 cells were treated with 10 mM lactate.

### Lactate concentration

In clinical samples, serum (*n* = 10) was isolated from the peripheral blood of healthy controls and sepsis patients by centrifugation at 3000 rpm for 15 min, and lactate levels were quantified using a colorimetric assay according to the manufacturer’s instructions (Solarbio, Beijing, China, BC2235). In animal experiments, lactate levels were measured in lung tissues collected from mice from different groups using the same colorimetric assay.

### Lipid peroxidation assays

Lipid peroxidation was determined by measuring glutathione peroxidase (GSH, Jiancheng Biology, Nanjing, China, A005-1-2) and Malondialdehyde (MDA, Jiancheng Biology, A003-1-2) levels using assay kits according to the manufacturer’s instructions.

### Reactive oxygen species

Reactive oxygen species (ROS) were measured using the fluorescent probe C11-BODIPY581/591 (ABP Biosciences, MD, USA, C258). Cells were incubated with C11-BODIPY (10 μM) at 37 °C for 30 min, washed, and analyzed by fluorescence microscopy. Oxidation of C11-BODIPY shifts fluorescence from red to green, with the ratio of green/red intensity indicating ROS levels.

### Enzyme-linked immunosorbent assay

The ELISA kits (Multi Sciences, Zhou Heng, China, EK106, EK101B, EK182, EK206, EK201B, EK282) were used to measure the levels of cytokines IL-6, IL-1β, and TNF-α in the serum of patients and mice according to the manufacturer’s protocols.

### Quantitative real-time polymerase chain reaction

Total RNA was extracted from tissues or cells using TRIzol reagent, followed by cDNA synthesis with reverse transcriptase. Quantitative real-time polymerase chain reaction (qRT-PCR) was performed using a Light Cycler apparatus (Roche, Rotkreuz, Switzerland). The primer sequences are presented in the Supplementary Material (Supplementary Table 1). Amplification was conducted on a real-time PCR system, and cycle threshold (Ct) values were recorded. Relative gene expression was calculated using the 2^(-ΔΔCt) method, normalized to β-actin.

### Lung wet-to-dry ratio

The left lungs of mice were harvested at 24 h and weighed to determine wet weight. The tissues were then dried at 65 °C for 48 h and reweighed to obtain dry weight. The wet-to-dry (W/D) ratio was calculated by dividing the wet weight by the dry weight.

### Histopathological analysis

Histopathological analysis was performed by isolating lung tissues and fixing them in 4% paraformaldehyde for 24 h at room temperature, followed by paraffin embedding. Tissue sections were stained with hematoxylin-eosin (HE) for histological examination. The severity of SI-ALI was evaluated using a semi-quantitative scoring system based on alveolar edema, hemorrhage, leukocyte infiltration, and alveolar wall thickness, with scores ranging from 0 (normal) to 3 (severe). Two researchers independently assessed each parameter in a double-blind manner to calculate the total lung injury score, ensuring unbiased and accurate evaluation.

### Western blot

Lung tissues were lysed with RIPA buffer and protease inhibitors (50:1, Beyotime, Shanghai, China). Protein levels were measured using a BCA kit (Bio-Rad, UK). Equal amounts of protein were separated by SDS-PAGE and transferred onto PVDF membranes. After blocking with 5% non-fat milk, membranes were incubated with primary antibodies overnight at 4 °C. For the details of antibodies, see Supplementary Table 2. Protein bands were visualized using a Tanon 5500 imaging system (Tanon, Shanghai, China). Relative protein expression was calculated by normalizing target protein grayscale values to β-actin or Tubulin as internal controls.

### Immunofluorescence

Lung tissue samples were sectioned into 10 μm slices using a cryostat microtome (HM550VP, MICROM, Germany). Sections were incubated with primary antibodies targeting specific proteins, such as LPCAT2 (1:500, Santa Cruz, Dallas, USA, sc-514354) and p-STAT1 (1:200, Cell Signaling Technology, Danvers, USA, 9167). Afterward, sections were treated with secondary antibodies, including Alexa 488-labeled goat anti-rabbit IgG (1:500, Abcam, Cambridge, UK, ab150077), for 1 h at 37 °C. Fluorescence images were acquired using a microscope (EVOS FL Auto, Thermo Fisher Scientific). Fields per sample were analyzed quantitatively using Image J (version 1.8.0, NIH, USA). In short, each RGB image was converted to 8-bit greyscale and then to binary using the “Dark Background” threshold in ImageJ. Using Image-Adjust-Threshold to select a suitable threshold. Analysis settings included area, area fraction, mean grey value, and integrated density. The mean integrated density per field was normalized by cell count [31]. The subcellular localization of the p-STAT1 protein was analyzed using a confocal laser scanning microscope (IX81-FV1000, Olympus). The method of calculating the nuclear-to-cytoplasmic ratio using ImageJ involves segmenting the nuclei and cytoplasm in the fluorescence images and measuring their respective fluorescence intensities. The nuclear-to-cytoplasmic ratio is then determined by calculating the ratio of nuclear fluorescence intensity to cytoplasmic fluorescence intensity.

### Cell viability

Cell viability was assessed using the Cell-Counting Kit-8 (CCK-8) assay (Beyotime, C0037) according to the manufacturer’s protocol. BEAS-2B cells were plated in 96-well plates at a density of 3500 cells per well. After treatment, the culture medium was replaced with fresh medium containing 10% CCK-8 solution at designated time points. The absorbance was measured at 450 nm using a multimode microplate reader.

### ATP concentration

Intracellular ATP in BEAS-2B cells was measured using an ATP assay kit (Beyotime, S0026). Cells were seeded in 6-well plates, treated with test compounds or PBS for 12 h, and washed with PBS. Cells were lysed with 200 µl ATP lysis buffer, centrifuged at 12,000 × g (4 °C, 5 min), and supernatants were collected. ATP levels were quantified using a microplate reader.

### siRNA transfection

BEAS-2B cells at 80% confluency in 6-well plates were transfected with a mixture of siRNA (50 nM) and Transfection Kit (Ribobio, Guangzhou, China, C10511-05). After 15 min incubation, the mixture was added to the cells and incubated at 37 °C. Medium was replaced after 6 h, and cells were harvested 48 h post-transfection for analysis. Efficiency was verified by Western blot (WB). The sequencing of siRNA is as follows: hsa-AARS1 (5’- CAAGAAGGCUGAAGAGAUUTT -3’ and 3’- AAUCUCUUCAGCCUUCUUGTT -5’), hsa-HDAC9 (5’- UGGAGAAGCAGAAGCAAUATT -3’ and 3’ – UAUUGCUUCUGCUUCUCCATT - 5’), and hsa-LPCAT2 (5’- CCAAUAAAGUCCGGAAUUUTT -3’ and 3’ – UUGGTIAAAUUCCGGACU - 5’).

### Plasmids

BEAS-2B cells were seeded in 6-well plates and cultured until 80% confluency. A transfection mixture was prepared by combining plasmid DNA with LipoTrans (Puzon, Beijing, China) in an Opti-MEM medium (Gibco^TM^, NY, USA). After 20 min incubation, the mixture was added to the cells and incubated at 37 °C. The medium was replaced after 6 h, and cells were harvested 48 h post-transfection. Overexpression efficiency was confirmed by WB analysis.

### Mutation

The generation of the LPCAT2 K375R mutation was performed by Puzon. Briefly, primers for site-directed mutagenesis were designed using a specialized online tool. PCR amplification was then conducted using the WT plasmid as the template to generate the desired point-mutated sequence. Following amplification, the PCR product was ligated into an appropriate vector, transformed into competent cells, and subsequently sequenced to confirm the presence of the mutation. The plasmid carrying the exogenous mutated gene was then transfected into BEAS-2B cells. Finally, the successful expression and incorporation of the mutated protein were verified through WB analysis.

### Lentiviral transduction

BEAS-2B cells were seeded in 24-well plates. When cells reached 50–60% confluence, they were transduced with concentrated lentivirus (MOI = 60) in the presence of Polybrene (Puzon). After 24 h, the transduction medium was replaced with complete growth medium. Transduced cells were selected with 2 μg/mL puromycin (Sigma-Aldrich) for 72 h to eliminate non-transduced cells.

### Co-immunoprecipitation assays

Approximately 50 mg tissues or 1×10^7^ cells were lysed in EBC buffer with protease/phosphatase inhibitors. 5% of the lysate was kept as an input control. For endogenous Co-immunoprecipitation (Co-IP), the rest was incubated with primary antibody overnight at 4 °C, then with protein A/G beads for 6 h at 4 °C. For exogenous Co-IP, it was incubated with rabbit anti-LPCAT2 (Santa Cruz, sc-514354), rabbit anti-Flag (Immunoway, Suzhou, China, YM3809), rabbit anti-STAT1 (Cell Signaling Technology, Danvers, USA, 14994), anti-L-Lactyl Lysine Rabbit mAB (PTMBIO, Wuhan, China, PTM-1401) and Anti-Acetyllysine Rabbit mAB (PTMBIO, PTM-101). After incubation, immune complexes were washed thrice with NETN buffer. Proteins were eluted under denaturing conditions and detected by WB.

### Lactylation proteomics

Lactylation proteomics was conducted by Hangzhou Cosmos Wisdom Biotech Co., Ltd. Proteins were extracted from tissue or body fluid samples and subsequently digested into peptides using trypsin. The resulting peptides were desalted and enriched. The enriched peptides were then analyzed by data-independent acquisition (DIA) on an Orbitrap Astral mass spectrometer. All DIA raw data obtained from liquid chromatography-tandem mass spectrometry (LC-MS/MS) analyses were processed. Quantitative results derived from DIA-NN were used as the basis for subsequent bioinformatics analysis workflows. The subcellular localization of differentially expressed proteins (DEP) was annotated using the WolF Psort software. Additionally, functional enrichment analyses were performed on the differentially expressed genes, employing the clusterProfiler package to conduct both Gene Ontology (GO) and Kyoto Encyclopedia of Genes and Genomes (KEGG) analyses [32]. The DEP in the heatmap and volcano plot were ranked based on their fold change (FC) values > 1.5, with all selected proteins/genes meeting the significance criterion of *p* < 0.05. The detailed methods for these are illustrated in Supplementary File 1.

### Proteomic analysis

For proteomic analysis of lung tissues, first, measure the protein content of each sample. Combine all labeled peptides and dry them via vacuum centrifugation. Load the peptides onto a C18 trap column for separation, and carry out liquid chromatography-tandem mass spectrometry on a timTOF Pro mass spectrometer. When identifying peptides, group proteins using the parsimony method, filter to achieve a 0.6% false discovery rate, and use only positively identified peptides for protein quantification. The DEP in the heatmap and volcano plot were ranked based on their fold change (FC) values > 1.5, with all selected proteins/genes meeting the significance criterion of *p* < 0.05. The detailed methods for these are illustrated in Supplementary File 1.

### Immunoprecipitation-Tandem Mass Spectrometry

To identify proteins that interact with LPCAT2, untargeted proteomic analysis was performed using Immunoprecipitation-Tandem Mass Spectrometry (IP-MS/MS). Co-IP assays were conducted in the lung epithelial cell line BEAS-2B using an anti-LPCAT2 antibody or IgG as a negative control. The proteins captured by the anti-LPCAT2 antibody were separated by SDS-PAGE. Subsequently, the proteins of interest were excised from the gel and analyzed by mass spectrometry.

### Transmission electron microscopy

Lung tissues were postfixed in 2% osmium tetroxide containing 1.6% potassium ferrocyanide in 0.1 M sodium buffer and sliced into 1 mm³ sections. After dehydration through a graded acetone series, samples were embedded in Eponate 812 resin. Ultrathin sections were stained with uranyl acetate and imaged using a transmission electron microscope (Hitachi, Tokyo, Japan, HT-7700). Sections were stained with uranyl acetate and lead citrate and then examined under a transmission electron microscope (TEM) at 80 kV. Images were captured to assess ultrastructural features.

### Chromatin immunoprecipitation assay

The chromatin immunoprecipitation (ChIP) assay was performed using a ChIP Kit (Beyotime, P2083S). 293 T cells were cross-linked with 1% formaldehyde for 15–20 min, and chromatin was extracted using a Chromatin Extraction Kit (Abcam, ab117152). After centrifugation and washing, chromatin was sheared to 200–500 bp fragments. Soluble chromatin was incubated with a STAT1-specific antibody or IgG. Immunoprecipitated DNA was purified and analyzed by qPCR to identify protein-DNA interaction. The sequences of the primers were as follows: forward: 5’ CCTCTATTCGGACCCATTTAGT 3’ and reverse: 5’ CTGGGTTTCTTGTCCCATATAA 3’.

### Kaplan-Meier survival analysis

After CLP surgery, survival was monitored for 96 h in each group. Survival status was coded as 0 (alive at the 96-h endpoint) and 1 (death within the observation period). Kaplan-Meier curves were plotted to compare cumulative survival probabilities across groups, and statistical differences were evaluated using the log-rank test (α = 0.05).

### Seahorse bioenergetic measurements

The extracellular acidification rate (ECAR) and oxygen consumption rate (OCR) were measured using an XF24 Extracellular Flux Analyzer (Seahorse Bioscience), with BEAS-2B cells seeded into XF24 microplates at 2 × 10⁴ cells/well and incubated overnight at 37 °C with 5% CO₂; for the LPS + PDK4-in group, cells were pre-treated with 30 μM PDK4-in for 24 h followed by 10 μg/mL LPS for 4 h, then for ECAR measurement, cells were washed and equilibrated in XF Base Medium supplemented with 2 mM L-glutamine for 1 h at 37 °C without CO₂ before injecting 25 mM glucose, 2 μM oligomycin (MedChemExpress, HY-N6782), and 50 mM 2-DG, while for OCR measurement, cells were similarly equilibrated and injected sequentially with 1 μM oligomycin, 1 μM FCCP [Carbonyl cyanide 4-(trifluoromethoxy)phenylhydrazone, MedChemExpress, HY-100410], and 1 μM each of rotenone (MedChemExpress, HY-B1756) / antimycin A (MKBIO, Shanghai, China, HY-MS0070).

### Generation of specific anti-LPCAT2-K375 antibody

To produce a specific antibody targeting lactylated K375 of LPCAT2, peptides A and B (with lactylated K375) and unmodified peptide C were designed. Peptides A and B were conjugated to Keyhole Limpet Hemocyanin (KLH) using Sulfosuccinimidyl 4-(N-Maleimidomethyl) cyclohexane-1-carboxylate (SMCC). The KLH-peptide A/B conjugates were mixed and used to immunize two rabbits with complete Freund’s adjuvant initially and incomplete Freund’s adjuvant for three boosts. After titer testing, two PBS boosters were administered. The collected serum was purified through a protein A column, enriched on a peptide A affinity column, and cross-reactive antibodies were removed using a peptide C column. The final antibody was validated for specificity to the lactylated epitope via ELISA and Western blotting.

### Statistical analysis

All statistical analyses were conducted using SPSS software (version 25.0; IBM Corp., Armonk, NY, USA). Data are presented as the mean ± SD. The t-test was used to compare the experimental data between the two groups. One-way ANOVA and Bonferroni post hoc comparisons were used to compare the experimental data from multiple groups. The log-rank test was used to determine the statistical significance of Kaplan–Meier survival curves. *P* < 0.05 was considered statistically significant.

## Results

### Lactate can aggravate SI-ALI

**Fig. 1:**
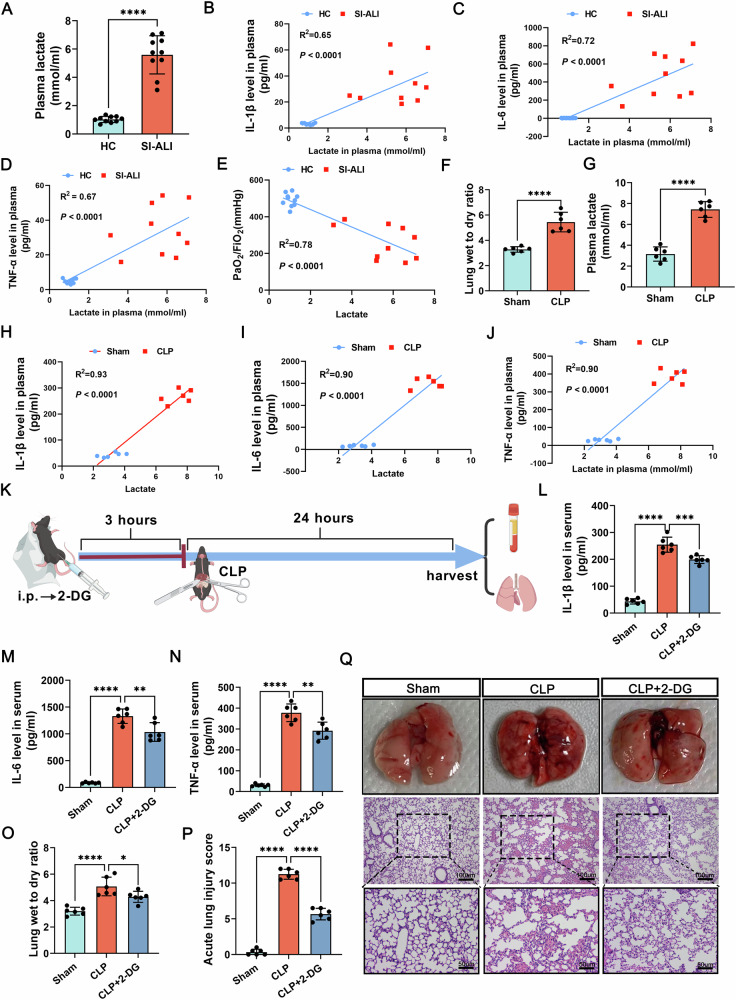
Inhibiting the lactate generation could alleviate SI-ALI. **A** Plasma lactate levels in clinical SI-ALI patients and healthy controls (HC). (*n* = 10). **B–D** The correlation between lactate and inflammatory factors (IL-1β, IL-6, and TNF-α) in plasma. **E** The correlation between lactate and PaO2/FiO2 ratio in plasma. (n = 10). **F** Wet-to-dry ratio of lungs in the sham group, compared to the CLP group. (*n* = 6). **G** Plasma lactate levels in the sham group and CLP group. (*n* = 6). **H–J** The correlation between plasma lactate and inflammatory factors (IL-1β, IL-6, TNF-α) in mice. (*n* = 6). **K** The schematic diagram of the experimental design, including the administration of 2-DG and the establishment of the CLP model. **L–N** Inflammatory factors (IL-1β, IL-6, and TNF-α) levels in plasma in the sham and CLP group with or without 2-DG administration. (*n* = 6). **O** Wet-to-dry ratio of lungs in the sham and CLP group with or without 2-DG administration. (*n* = 6). **P, Q** Acute lung injury score in the sham and CLP group with or without 2-DG administration. (*n* = 6, up: scale bar =100 μm; down: scale bar =50 μm). Data are presented as mean ± SD. *p < 0.05, **p < 0.01, ***p < 0.001, and ****p < 0.0001. Statistical significance was determined by one-way ANOVA, Student’s t-test or log-rank test as appropriate. Each experiment was conducted with ten or six independent biological replicates.

Elevated plasma lactate is clinically associated with worsened outcomes and multi-organ dysfunction in sepsis [33]. Our study revealed significantly higher lactate levels in SI-ALI patients compared to healthy controls (Fig. 1A). We measured plasma levels of inflammatory factors (IL-1β, IL-6, and TNF-α) and assessed lung injury severity using the arterial oxygen partial pressure (PaO2) / fraction of inspiration O2 (FiO2) ratio. The PaO2/FiO2 ratio was derived from the initial arterial blood gas analysis results of sepsis patients who met Sepsis-3 criteria within 24 h of admission, utilizing the values of PaO2 and FiO2. This ratio serves as a crucial benchmark for assessing the SI-ALI [34]. Our analysis revealed a positive correlation between plasma lactate and inflammatory factor concentrations (Fig. 1B–D), suggesting that elevated lactate may contribute to systemic inflammation. Furthermore, SI-ALI patients with elevated lactate exhibited significantly worse lung function, as indicated by a lower PaO2/FiO2 ratio (Fig. 1E).

In CLP mice, they exhibited significantly higher lactate levels and more severe lung damage compared to the sham group (Fig. 1F, G). Additionally, a positive correlation was observed between plasma lactate and inflammatory factor concentrations in these mice (Fig. 1H–J). To further explore the relationship between lactate and SI-ALI, 2-DG, a pan-glycolysis inhibitor, which could reduce the production of lactate, was utilized for CLP and sham group mice. The scientific drawing materials were created with BioGDP.com (Fig. 1K) [35]. This treatment markedly reduced inflammatory factor levels (Fig. 1L–N), decreased the lung W/D ratio, and attenuated acute lung injury scores (Fig. 1O–Q). These results suggest that inhibiting lactate production can effectively mitigate systemic inflammation and alleviate SI-ALI.

### PDK4-dependent glycolytic reprogramming promotes lactate accumulation in SI-ALI

**Fig. 2:**
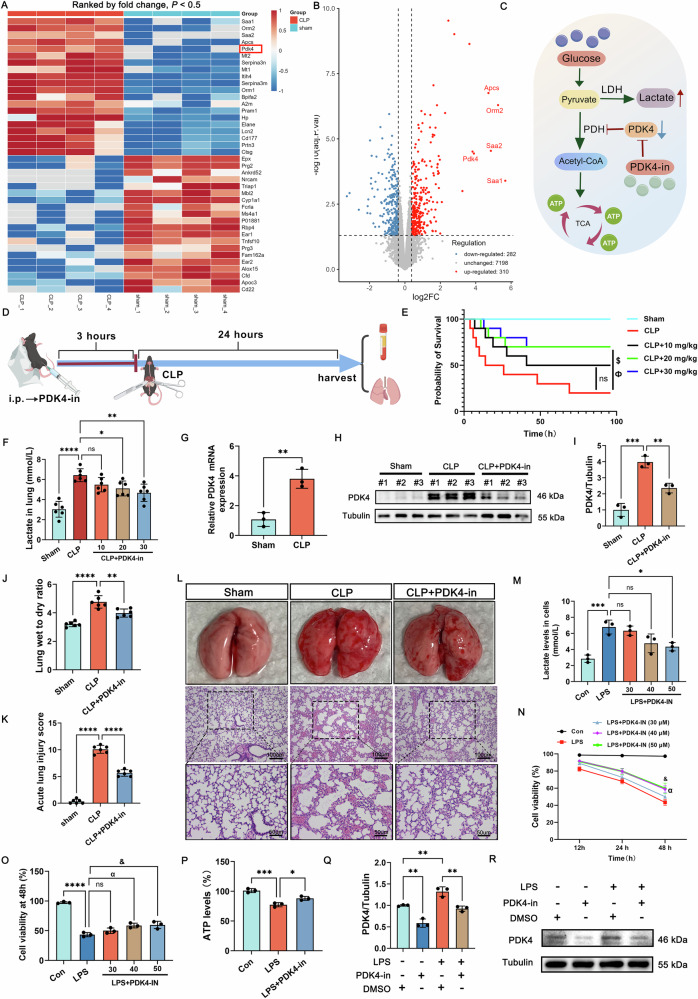
The elevated expression of PDK4 plays a pivotal role in driving excessive lactate accumulation in lung tissue during sepsis. **A, B** The heatmap and volcano plot of proteomic analysis. (*n* = 4). **C** The schematic diagram depicting PDK4 inhibition-mediated suppression of lactate generation in SI-ALI. **D** The schematic diagram of the experimental design, including the administration of PDK4-in and the establishment of the CLP model. **E** The Kaplan-Meier survival analysis of the sham and CLP group with or without PDK4-in administration. (*n* = 10). **F** Lactate levels in the lung tissues of the sham and CLP group with or without PDK4-in administration. (*n* = 6). **G** The levels of PDK4 mRNA in lung tissues in the sham and CLP group. (*n* = 3). **H, I** The expression of PDK4 in the sham and CLP group with or without PDK4-in administration. (*n* = 3). **J** Wet-to-dry ratio of lungs in the sham and CLP group with or without PDK4-in administration. (*n* = 6). **K, L** Acute lung injury score in the sham and CLP group with or without PDK4-in administration. (*n* = 6, Up, scale bar =100 μm; down, scale bar =50 μm). **M** Lactate levels in the control and LPS-treated groups with or without PDK4-in treatment. (*n* = 3). **N, O** Comparative analysis of cell viability between control and LPS-treated groups with or without PDK4-in treatment at 48 h. (*n* = 3). **P** The levels of ATP in control and LPS-treated groups with or without PDK4-in treatment in BEAS-2B cells. (*n* = 3). **Q, R** The expression of PDK4 in control and LPS-treated groups with or without PDK4-in treatment in BEAS-2B cells. (*n* = 3). Data are presented as mean ± SD. ^$^*p* < 0.05, ^Ф^*p* < 0.05, ^α^*p* < 0.0001, ^&^*p* < 0.0001^,^ *p < 0.05, **p < 0.01, ^*^**p < 0.001, and ****p < 0.0001. $: CLP VS. CLP + 20 mg/kg, Ф: CLP VS. CLP + 30 mg/kg, α: LPS VS. LPS + 40 μM, &: LPS VS. LPS + 50 μM. Statistical significance was determined by one/two -way ANOVA. Each experiment was conducted with six or three independent biological replicates, except the proteomic analysis (*n* = 4) and Kaplan-Meier survival analysis (*n* = 10).

The core pathways of lactate production include glycolysis and pyruvate metabolism, with key enzymes such as lactate dehydrogenases (LDHA/LDHB) [36], pyruvate dehydrogenase kinases (PDK1-4) [37], and monocarboxylate transporters (MCT1-4) [38], et al. In proteomic analysis, the heatmap shows PDK4 is the only protein with statistically significant upregulation in lactate-related expression (Fig. 2A, B). PDK4-in blocks PDK4 to reactivate pyruvate dehydrogenase (PDH), shifting pyruvate conversion to acetyl-CoA, enhancing the tricarboxylic acid (TCA) cycle and ATP production while reducing lactate generation (Fig. 2C). Treatment with PDK4-in significantly enhanced survival rates in CLP mice (Fig. 2D, E), while reducing lactate accumulation and PDK4 expression in septic lung tissues (Fig. 2F–I). Additionally, PDK4-in treatment significantly decreased the lung W/D ratio and attenuated acute lung injury scores in CLP mice (Fig. 2J–L).

In vitro studies, we found that LPS can significantly increase lactate generation, and PDK4-in significantly attenuated lactate accumulation and mitigated lactate-induced cellular damage and death (Fig. 2M–O). Furthermore, PDK4-in suppressed glycolytic metabolic reprogramming, as indicated by restored ATP levels (Fig. 2P). ECAR and OCR were also employed to quantify glycolysis and mitochondrial respiration respectively. LPS-enhanced glycolysis and suppressed mitochondrial respiration were partly reversed by PDK4 inhibition (Fig. S1A–D), while LPS-upregulated PDK4 expression was markedly reduced by PDK4 inhibitor treatment (Fig. 2Q, R). Collectively, these findings demonstrate that PDK4 may play a key role in lactate production in SI-ALI, driving metabolic reprogramming and contributing to disease progression.

### LPCAT2-K375 lactylation may play an important role in the pathogenesis of SI-ALI

**Fig. 3:**
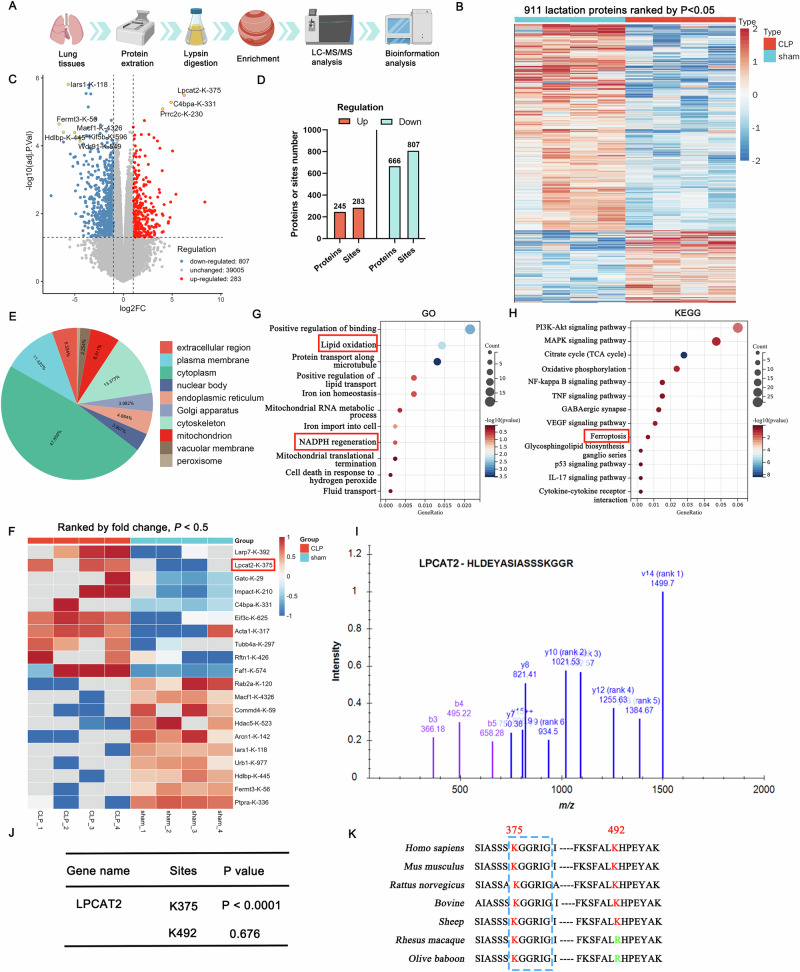
The elevated lactylation level of LPCAT2 is one of critical factors contributing to SI-ALI. **A** The schematic diagram of lactylation proteomics. **B, C** The heatmap and volcano plot of lactylation proteomics. (*n* = 4). **D, E** The number and distribution of proteins and sites of lactylation in the lung tissues in SI-ALI. **F** LCPAT2-K375 was significantly upregulated in SI-ALI (The first protein was excluded due to non-conserved amino acid sequences across species). **G, H** GO and KEGG enrichment analyses of lactylation proteomics. **I** A representative LPCAT2 peptide with lactylation modification at lysine 375 (K375). **J** The *p*-values of the lactylation sites in LPCAT2. **K** Protein multiple sequence alignment analysis revealed that only the K375 site was relatively conserved among species.

To identify non-histone lactylated proteins in SI-ALI tissues, we performed lactylome sequencing, revealing 911 lactylated proteins (283 upregulated and 807 downregulated sites) (Fig. 3A–E). This extensive profiling highlights the widespread nature of lactylation in the context of SI-ALI.

Among these proteins, the heatmap analysis revealed that LPCAT2-K375 was significantly upregulated in SI-ALI (Fig. 3F). Previous studies have demonstrated that LPCAT2 primarily mediates ferroptosis progression [39, 40]. GO and KEGG analyses revealed that non-histone lactylation modifications in SI-ALI significantly influence ferroptosis-related pathways, (Fig. 3G, H, Supplementary Table 3). Sequencing identified two potential lactylation sites in LPCAT2, but only K375 showed significant lactylation and was conserved across various species (Fig. 3I–K). Given lactylation analysis revealed marked LPCAT2-K375 lactylation upregulation in SI-ALI tissues, suggesting it is one of the critical roles in exacerbating pulmonary injury.

### The level of LPCAT2-K375 lactylation is significantly increased in SI-ALI

**Fig. 4:**
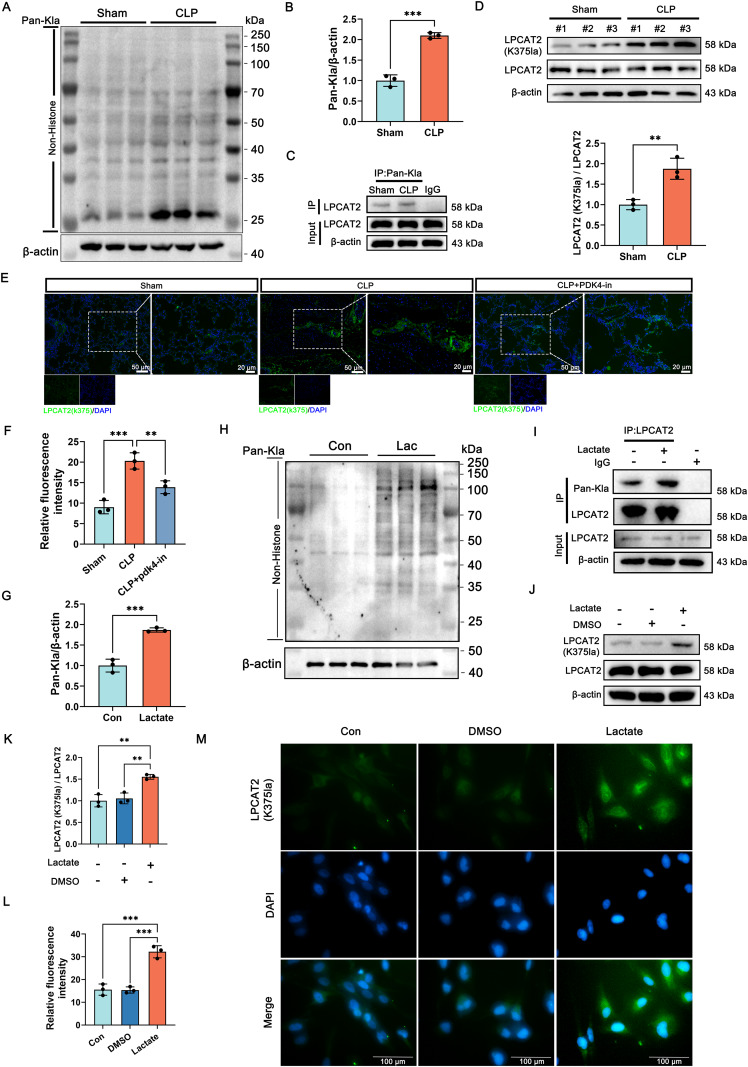
Lactate could upregulate the LPCAT2-K375 lactylation. **A, B** The expression of lactylation in lung tissues between the sham and CLP groups. (*n* = 3). **C** Co-IP result showed the interaction of Pan-Kla with LPCAT2 in lung tissues in the sham and CLP group. (*n* = 3). **D** The expression of LPCAT2-K375 and LPCAT2 in the sham and CLP group. (*n* = 3). **E, F** Immunofluorescence images and quantitative analysis of LPCAT2-K375 expression in the lung in the sham and CLP group with or without PDK4-in administration. (*n* = 3, left: scale bar =50 μm, right: scale bar =20 μm). **G, H** The expression of lactylation in BEAS-2B cells between the Con and Lac groups. (*n* = 3). **I** Co-IP analysis demonstrated changes in LPCAT2 lactylation levels in BEAS-2B cells, comparing control and lactate-treated groups. **J, K** The expression of LPCAT2-K375 and LPCAT2 in BEAS-2B cells with or without lactate treatment. (*n* = 3). **L, M** Immunofluorescence images showed LPCAT2-K375 expression in BEAS-2B cells with or without lactate treatment. (*n* = 3, scale bar =100 μm). Data are presented as mean ± SD. **p < 0.01, ***p < 0.001. Statistical significance was determined by one-way ANOVA or Student’s *t*-test as appropriate. Each experiment was conducted with three independent biological replicates.

In vivo experiments, WB showed markedly elevated lactylation in SI-ALI tissues versus sham (Fig. 4A, B). Co-IP and WB showed upregulated lactylated LPCAT2-K375 in the CLP versus sham group (Fig. 4C, D). Immunofluorescence staining results also showed a significant increase in LPCAT2-K375 expression in SI-ALI tissues, further supporting its potential involvement in the pathogenesis of SI-ALI (Fig. 4E, F).

In vitro studies, the levels of lactylation were significantly elevated in BEAS-2B cells after treatment with lactate (Fig. 4G, H). Co-IP experiments confirmed the presence of lactylation on LPCAT2 in BEAS-2B cells (Fig. 4I, Fig. S4A). Furthermore, lactate treatment upregulated the expression of LPCAT2-K375 (Fig. 4J, K), which was validated by immunofluorescence staining (Fig. 4L, M). These findings collectively indicate that lactate-induced LPCAT2-K375 lactylation drives SI-ALI pathogenesis, highlighting its potential as a therapeutic target.

### Lactate aggravates ferroptosis in epithelial cells by suppressing SLC7A11

**Fig. 5:**
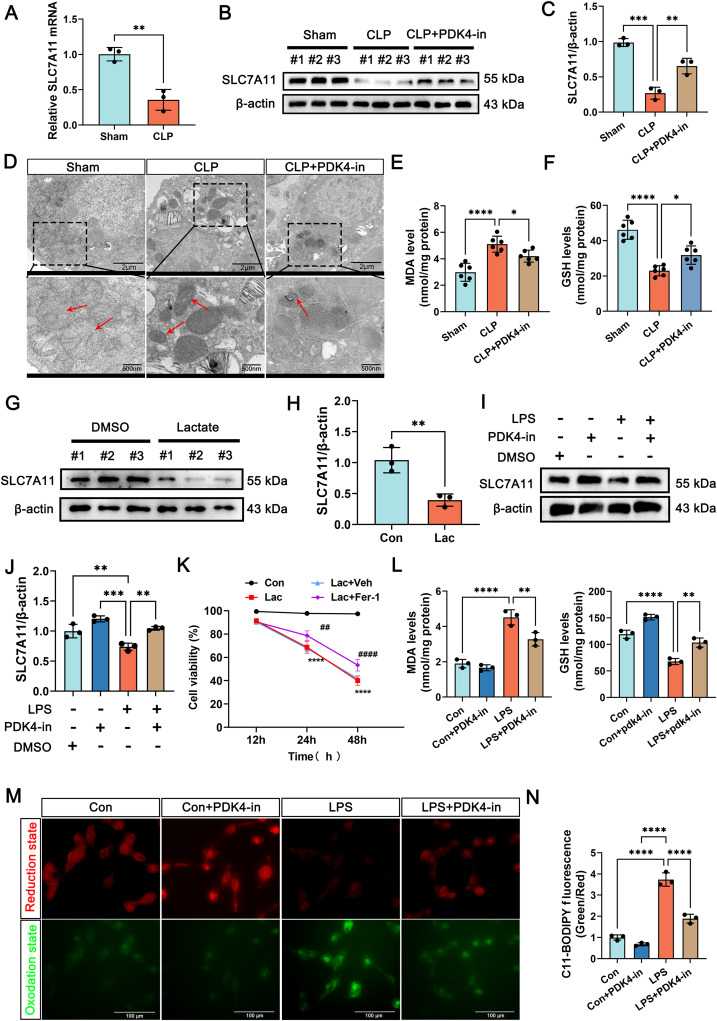
Lactate aggravates ferroptosis in epithelial cells by suppressing SLC7A11. **A** The levels of SLC7A11 mRNA in lung tissues in the sham and CLP group. (*n* = 3). **B, C** The expression of SLC7A11 in the sham and CLP group with or without PDK4-in administration. (*n* = 3). **D** The representative images of TEM showed mitochondrial changes in lung tissues in the sham and CLP group with or without PDK4-in administration. (*n* = 3, up: scale bar =2 μm; down: scale bar =500 nm). **E, F** The levels of MDA and GSH in the sham and CLP group with or without PDK4-in administration. (*n* = 6). **G, H** The expression of SLC7A11 in control and LPS-treated groups with or without PDK4-in treatment in BEAS-2B cells. (*n* = 3). **I, J** The expression of SLC7A11 in control and lactate-treated groups with or without PDK4-in treatment in BEAS-2B cells. (*n* = 3). **K** Comparative analysis of cell viability was conducted among different groups of BEAS-2B cells at the 48-h time point. (*n* = 3, **#**: Con VS. Lac+Fer-1, *: Con VS. Lac). **L** The levels of MDA and GSH in control and LPS-treated groups with or without PDK4-in treatment in BEAS-2B cells. (*n* = 3). **M, N** Representative images and quantitative analysis of C11-BODIPY 581/591 staining in control and LPS-treated groups with or without PDK4-in treatment in BEAS-2B cells. (*n* = 3, scale bar =100 μm). Data are presented as mean ± SD. ^**##**^*p* < 0.01, ^**####**^*p* < 0.0001, *p < 0.05, **p < 0.01, ***p < 0.001, and ****p < 0.0001. Statistical significance was determined by one-way ANOVA or Student’s *t*-test as appropriate. Each experiment was conducted with six or three independent biological replicates.

To verify that lactate and ferroptosis aggravate SI-ALI, we treated CLP mice with lactate and the novel ferroptosis inhibitor UAMC-3203. The results indicated that lactate exacerbated ferroptosis in SI-ALI, while UAMC-3203 mitigated SI-ALI (Fig. S2A–F). The levels of SLC7A11 were downregulated under high lactate conditions but restored by PDK4-in (Fig. 5A–C). Mitochondrial dysfunction in CLP tissues was also improved after PDK4-in treatment (Fig. 5D), which also significantly reduced lipid peroxide accumulation (Fig. 5D–F). These findings suggest that inhibiting PDK4 could attenuate ferroptosis.

In vitro experiments, lactate significantly suppressed SLC7A11 expression (Fig. 5G, H), whereas PDK4-in treatment reversed this effect (Fig. 5I, J). The ferroptosis inhibitor also enhanced the viability of BEAS-2B cells under high lactate conditions (Fig. 5K), indicating that ferroptosis occurs under high lactate environment. This phenomenon was also observed in MLE12 cells: we found that lactate could upregulate the level of lipid peroxidation in MLE12 cells, while the ferroptosis inhibitor fer-1 could downregulate it (Fig. S3A–D). In addition, PDK4-in treatment elevated GSH and decreased MDA / ROS levels in the LPS group (Fig. 5L–N). Collectively, these results demonstrate that LPCAT2 mediates ferroptosis by regulating the expression of SLC7A11, and PDK4-in can effectively alleviate ferroptosis by restoring SLC7A11 expression. These insights highlight the potential therapeutic benefits of targeting PDK4 in SI-ALI.

### LPCAT2-K375 mutation suppresses ferroptosis in epithelial cells by suppressing SLC7A11

**Fig. 6:**
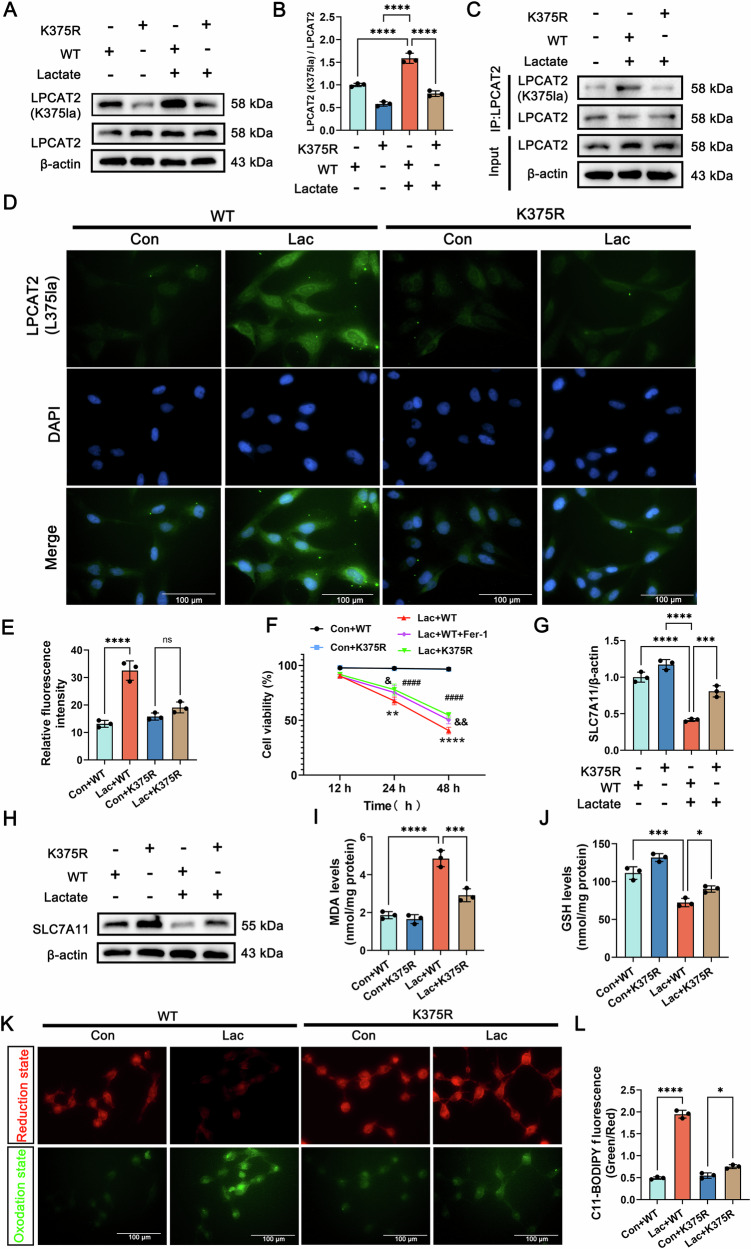
The K375 mutation in LPCAT2 attenuates ferroptosis in epithelial cells. **A, B** The expression of LPCAT2-K375 and LPCAT2 in the WT and K375R group with or without lactate treatment in BEAS-2B cells. (*n* = 3). **C** Co-IP result showed the expression of LPCAT2-K375 and LPCAT2 in the WT and K375R group with or without lactate treatment in BEAS-2B cells. (*n* = 3). **D, E** Immunofluorescence images and quantitative analysis of LPCAT2-K375 expression in the WT and K375R group with or without lactate treatment in BEAS-2B cells. (*n* = 3, scale bar =100 μm). **F** Comparative analysis of cell viability among different groups in BEAS-2B cells at 48 h. (*n* = 3, #: Con+WT VS. Lac+WT, *: Lac+WT VS. Lac+K375R, &: Lac+WT VS. Lac+WT+Fer-1). **G, H** The expression of SLC7A11 in the WT and K375R group with or without lactate treatment in BEAS-2B cells. (*n* = 3). **I, J** The levels of MDA and GSH in the WT and K375R group with or without lactate treatment in BEAS-2B cells. (*n* = 3). **K, L** Representative images and quantitative analysis of C11-BODIPY 581/591 staining in the WT and K375R group with or without lactate treatment in BEAS-2B cells. (*n* = 3, scale bar =100 μm). Data are presented as mean ± SD. ^**####**^*p* < 0.0001, ^&^ *p* < 0.05, ^& &^ *p* < 0.01, *p < 0.05, ^**^p < 0.01, ***p < 0.001, and ****p < 0.0001. Statistical significance was determined by one-way ANOVA or Student’s *t*-test as appropriate. Each experiment was conducted with three independent biological replicates.

To investigate whether LPCAT2-K375 lactylation promotes ferroptosis by regulating SLC7A11, we created an LPCAT2-K375 mutant (LPCAT2-K375R) using lysine-to-arginine site-directed mutagenesis. BEAS-2B cells were transfected with lentivirus carrying LPCAT2-WT or LPCAT2-K375R mutants. WB and Co-IP showed that LPCAT2-K375 decreased in BEAS-2B cells expressing LPCAT2-K375R versus WT (Fig. 6A–C). Immunofluorescence staining further confirmed these findings (Fig. 6D, E).

Next, we evaluated the impact of the LPCAT2-K375R on cell viability. LPCAT2-K375R expression enhanced BEAS-2B cell viability, indicating ferroptosis suppression (Fig. 6F). WB analysis confirmed upregulated SLC7A11 expression in LPCAT2-K375R cells versus WT (Fig. 6G, H). Furthermore, these LPCAT2-K375R cells demonstrated significantly increased GSH and decreased MDA / ROS levels (Fig. 6I–L). In summary, our findings show that LPCAT2-K375 regulates ferroptosis via SLC7A11. K375 mutation inhibits ferroptosis by increasing SLC7A11 and reducing lipid peroxidation. These results underscore the key role of LPCAT2-K375 in ferroptosis and its therapeutic potential for SI-ALI.

### LPCAT2-K375 transcriptionally represses SLC7A11 by promoting STAT1 phosphorylation and nuclear translocation

**Fig. 7:**
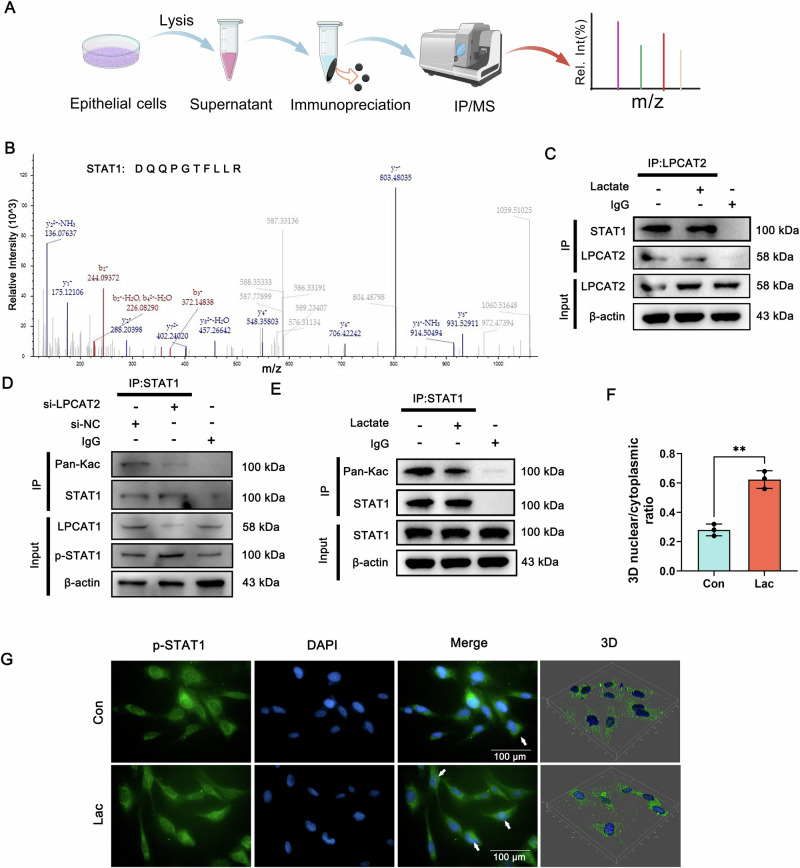
LPCAT2-K375 suppresses SLC7A11 expression by inhibiting STAT1 acetylation and promoting STAT1 phosphorylation and nuclear translocation. **A** Flowchart illustrating the experimental procedure of IP-MS/MS analysis. **B** Mass spectrometry analysis identified STAT1 as one of the highly abundant proteins enriched by anti-LPCAT2 antibody. **C** Co-IP result showed the interaction of LPCAT2 with STAT1 between control and lactate-treated groups in BEAS-2B cells. **D** Co-IP analysis demonstrated that LPCAT2 could mediate the STAT1 acetylation levels. **E** Co-IP analysis demonstrated changes in STAT1 acetylation levels in BEAS-2B cells, comparing control and lactate-treated groups. **F, G** Immunofluorescence images and 3D model analysis demonstrated p-STAT1 distribution in BEAS-2B cells with or without lactate treatment. (*n* = 3, scale bar =100 μm). Data are presented as mean ± SD. Statistical significance was determined by Student’s *t*-test. Each experiment was conducted with three independent biological replicates.

To investigate how LPCAT2-K375 regulated the expression of SLC7A11, we performed IP/MS and identified STAT1 as an interacting partner (Fig. 7A, B). STAT1 has been well-documented as a transcriptional repressor of SLC7A11 expression [41, 42]. Molecular docking studies were performed to indicate that LPCAT2 and STAT1 interact through hydrogen bonding (Fig. S4B–D). The Co-IP assays also demonstrated that LPCAT2 can indeed bind to STAT1 (Fig. 7C).

Prior research has shown that LPCAT2 possesses acetylase activity [40]. Therefore, we conducted experiments and found that downregulation of LPCAT2 reduces the acetylation levels of STAT1 (Fig. 7D). Interestingly, we also observed that STAT1 acetylation was downregulated under lactate-treated conditions (Fig. 7E), suggesting that this activity is inhibited in the presence of lactate. Decreased acetylation of STAT1 increases its phosphorylation. We then performed confocal fluorescence microscopy and 3D modeling, which revealed that lactate promotes STAT1 phosphorylation and its nuclear translocation (Fig. 7F). We also assessed p-STAT1 levels in LPCAT2-K375R cells, and results showed that the K375 mutant significantly suppressed STAT1 phosphorylation under high lactate (Fig. S4E, F). CHIP assay demonstrated a transcriptional regulatory interaction between STAT1 and the SLC7A11 promoter (Fig. S4G). A previous study reported that SLC7A11 could also be regulated by PRMT1 [40]. However, we found that the levels of SLC7A11 did not downregulate significantly after treating cells with a PRMT1 inhibitor under high-lactate conditions (Fig. 3E, F). In addition, ACSL4 is one of the key regulatory molecules of ferroptosis [39]. Nevertheless, treatment with the ACSL4 inhibitor AS-252424 failed to significantly reduce lipid peroxidation in LPCAT2-K375R cells under high lactate (Fig. S3G, H). These findings suggest the LPCAT2-STAT1-SLC7A11 axis is likely the primary driver of lipid peroxidation in LPCAT2-regulated pathways under high lactate, and show LPCAT2-K375 lactylation represses SLC7A11 transcription by promoting STAT1 phosphorylation and nuclear translocation, uncovering a new mechanism for LPCAT2-K375 in regulating ferroptosis via SLC7A11.

### AARS1/HDAC9 is the lactyltransferase/ delactylase of LPCAT2-K375

**Fig. 8:**
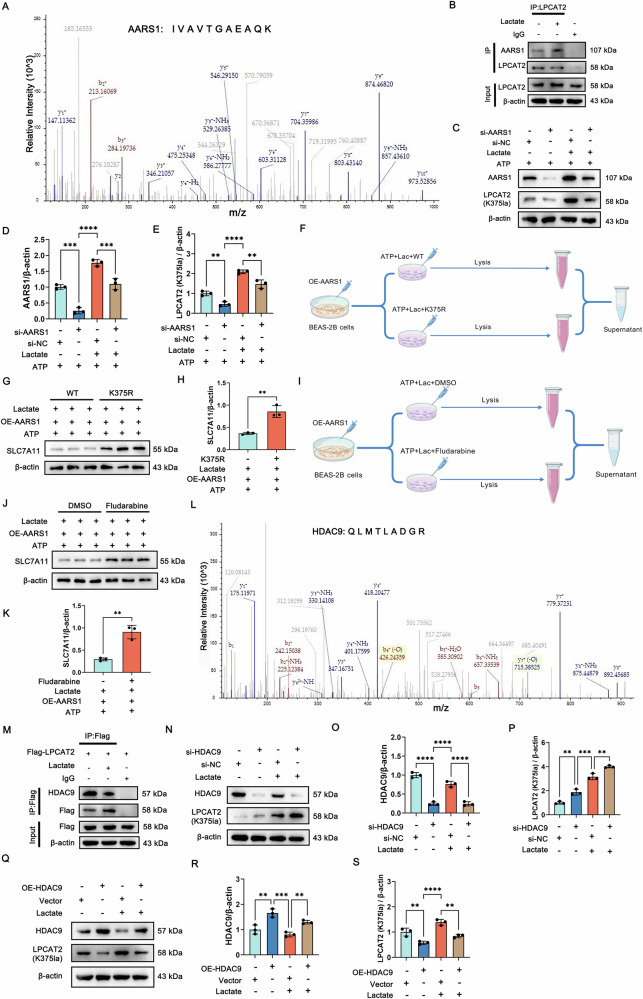
AARS1/HDAC9 is the lactyltransferase/delactylase of LPCAT2-K375. **A** Mass spectrometry analysis identified AARS1 as one of the highly abundant proteins enriched by anti-LPCAT2 antibody. **B** Co-IP result showed the interaction of AARS1 with LPCAT2 between control and lactate-treated groups in BEAS-2B cells. **C–E** The expression levels of AARS1 and LPCAT2-K375 following AARS1 knockdown. (*n* = 3). **F** The schematic diagram for the rescue experiment of LPCAT2-K375. **G, H** The expression of SLC7A11 in the WT and K375R group with or without overexpression of AARS1 treatment in BEAS-2B cells. (*n* = 3). **I** The schematic diagram for the rescue experiment of STAT1. **J, K** The expression of SLC7A11 in BEAS-2B cells with or without fludarabine treatment. **L** Mass spectrometry analysis identified HDAC9 as one of the highly abundant proteins enriched by anti-LPCAT2 antibody. **M** Co-IP result showed the interaction of HDAC9 with LPCAT2 between control and lactate-treated groups in BEAS-2B cells. **N–P** The expression levels of HDAC9 and LPCAT2-K375 following HDAC9 knockdown. (*n* = 3). **Q–S** The expression levels of HDAC9 and LPCAT2-K375 lactylation following HDAC9 overexpression. (*n* = 3). Data are presented as mean ± SD. **p < 0.01, ***p < 0.001, and ****p < 0.0001. Statistical significance was determined by one-way ANOVA or Student’s *t*-test as appropriate. Each experiment was conducted with three independent biological replicates.

IP/MS identified AARS1 as the lactyltransferase mediating LPCAT2-K375 lactylation (Fig. 8A), which has recently been reported to function as a lactyltransferase in the presence of lactate and ATP [30, 43]. Molecular docking studies were also performed to indicate that LPCAT2 and AARS1 interact through hydrogen bonding (Fig. S5A–C). Co-IP experiment confirmed that there is a direct interaction between LPCAT2 and AARS1 (Fig. 8B). AARS1 upregulation in CLP (Fig. S5D–F) mediated LPCAT2-K375 lactylation, and its knockdown significantly reduced LPCAT2-K375 levels (Fig. 8C–E). Overexpression of AARS1 upregulated SLC7A11 expression via LPCAT2-K375 lactylation (Fig. 8F–H). Additionally, to examine whether STAT1 phosphorylation modulates SLC7A11 expression, we overexpressed AARS1 and found that SLC7A11 levels were significantly increased after treatment with fludarabine, an inhibitor of STAT1 phosphorylation (Fig. 8I–K). In summary, our findings demonstrate that AARS1 is the lactyltransferase responsible for the lactylation of LPCAT2-K375.

IP/MS further identified HDAC9 as the delactylase regulating LPCAT2-K375 lactylation (Fig. 8L). Molecular docking analysis reveals that LPCAT2 interacts with HDAC9 via hydrogen bonding (Fig. S6A–C). Co-IP experiment confirmed a direct interaction between LPCAT2 and HDAC9 (Fig. 8M). We also found that the expression levels of HDAC9 were significantly downregulated in the CLP group (Fig. S6D–F).

To further investigate the function of HDAC9, we knocked down its expression and the results showed that the expression of LPCAT2-K375 was significantly upregulated in lactate (Fig. 8N–P). Additionally, overexpression (OE) of HDAC9 significantly reduced LPCAT2-K375 lactylation in lactate-treated cells. This further supports the role of HDAC9 in negatively regulating LPCAT2-K375 lactylation. Previous studies indicated that HDAC9 can facilitate STAT1 deacetylation and increase its phosphorylation [44]. Given the observed downregulation of HDAC9 expression in SI-ALI, we further investigated p-STAT1 expression under high-lactate conditions. The results demonstrated a significant increase in phosphorylated STAT1 levels (Fig. S6G, H). These findings suggest that STAT1 phosphorylation is predominantly influenced by the lactylation status of LPCAT2-K375.

## Discussion

In this study, we demonstrated that high expression of PDK4 is a critical factor driving metabolic reprogramming in SI-ALI, leading to elevated lactate levels. Lactate could increase the lactylation of LPCAT2, which is facilitated by AARS1 and reduced by HDAC9. LPCAT2 lactylation impairs its acetyltransferase activity, increasing STAT1 phosphorylation and transcriptional repression of SLC7A11. This downregulation of SLC7A11 induces ferroptosis in epithelial cells and exacerbates SI-ALI. Knocking down PDK4 or mutating LPCAT2-K375 can alleviate ferroptosis of epithelial cells and mitigate SI-ALI. In summary, our study reveals a novel mechanism linking metabolic reprogramming and lactate overproduction to ferroptosis in SI-ALI via LPCAT2-K375 lactylation.

In septic patients, blood lactate levels increase with ALI progression and correlate with lung damage severity [45]. This finding is consistent with our results, which indicate a positive correlation between plasma lactate and inflammatory factor concentrations, as well as a decline in lung function. Nevertheless, the precise mechanism underlying lactate generation remains elusive in SI-ALI. In this study, we discovered that high expression of PDK4 might hold the key to lactate accumulation in SI-ALI. The PDK-lactic acid axis is regarded as a critical link between metabolic reprogramming and some diseases [46]. While we did not detect significant differential expression of PDK1 or PDK3 in our sequencing data, this may be attributed to differences in the environmental conditions and tissues, which can lead to distinct patterns of gene expression. In the context of sepsis, HIF-1α functions as a mediator in modulating the expression of PDK4 [47]. The upregulated PDK4 can trigger metabolic reprogramming, which is consistent with the results of our ECAR and OCR assays. Specifically, the activated PDK4 exerts an inhibitory effect on the PDH, impeding the TCA cycle and consequently redirecting pyruvate towards lactate production [48]. Previous studies showed that dichloroacetate, a PDK inhibitor, reduces lactate levels and improves survival in heart models by restoring PDH activity [49]. In this study, the PDK4 inhibitor PDK4-in [50], significantly reduced lactate production and ameliorated SI-ALI. The aforementioned findings suggest that PDK4 could potentially serve as a viable target for the treatment of SI-ALI.

Through lactylation sequencing, we identified LPCAT2 as one of the most prominently lactylated proteins under lactate-enriched conditions. LPCAT3 had also been shown to be associated with ferroptosis [51]. In our study, we did not detect significant differential expression in other LPCATs except for LPCAT2. According to the previous study, LPCAT2 mediated the ferroptosis PRMT1/SLC7A11 axis [40]. However, IP-MS analysis of LPCAT2 in BEAS-2B cells showed no significant PRMT1 enrichment, and PRMT1 inhibition did not cause significant downregulate in SLC7A11 levels under high-lactate conditions, suggesting PRMT1 may not be the key regulator of SLC7A11 in this condition. We further demonstrated that lactylated LPCAT2 could enhance STAT1 phosphorylation, which transcriptionally represses SLC7A11 expression, thereby driving lipid peroxidation. However, due to the lack of direct evidence like in vitro kinase structure knockout assays, the conclusion is constrained, so the solid arrow in the graphical abstract has been changed to a dashed one. Moreover, treatment with the ACSL4 inhibitor AS-252424 failed to significantly reduce lipid peroxidation in LPCAT2-K375R cells under high lactate. These findings suggest that, in the oxidative stress model examined here under high lactate levels, the LPCAT2-STAT1-SLC7A11 axis may be the primary driver of lipid peroxidation. Collectively, these findings disclosed a novel non-histone mechanism by which lactate triggers ferroptosis in epithelial cells and aggravates SI-ALI.

Lactylation, a lactate-derived PTM, covalently modifies lysine residues [52], which could alter enzyme conformation or substrate binding. For instance, a previous study reported that lactylation of fructose-bisphosphate ALDOA could inhibit its enzymatic activity [53]. In the present study, we discovered that the acetylase activity of LPCAT2 was downregulated following lactylation. As the potential downstream effector of LPCAT2, STAT1 exerts its transcriptional function after phosphorylation [54–56]. As for how reduced STAT1 acetylation facilitates STAT1 phosphorylation, previous studies have shown that the acetylation sites of STAT1 (K685 and K703) are adjacent to the phosphorylation site (Y701). There is spatial competition and functional antagonism between these sites [57]. After deacetylation, Y701 is more readily recognized by phosphatases, which facilitates STAT1 phosphorylation. Therefore, in our study, the reduced acetylase activity of LPCAT2 led to an increase in STAT1 phosphorylation and repressed the expression of SLC7A11.

In our research, we identified AARS1 as the potential lactyltransferase of LPCAT2. Previous studies have demonstrated that AARS1 acts as a lactate sensor, mediating lysine lactylation [30, 58]. Briefly, AARS1 binds to lactate and ATP, forming a lactate-AMP intermediate and transferring the activated lactate group to a lysine residue on LPCAT2-K375 [30]. Under physiological conditions, lactate concentrations typically exceed 0.5 mM under basal conditions [59]. In other words, lactylation can also occur under physiological conditions, as demonstrated in our results. Our IP/MS analysis also identified HDAC9 as a potential delactylase, in addition to its previously reported deacetylase activity [60, 61].

In SI-ALI, HDAC9 expression was markedly decreased, and knockdown of HDAC9 significantly elevated the lactylation level of LPCAT2-K375. Moreover, overexpressing HDAC9 would downregulate the lactylation of LPCAT2-K375. Interestingly, HDAC9 was also reported to be involved in reducing the acetylation of STAT1 and increasing STAT1 phosphorylation [62]. Nevertheless, the levels of HDAC9 were downregulated in SI-ALI. If there was a significant mediating effect between HDAC9 and STAT1, the expression of STAT1 phosphorylation should have been downregulated. Instead, the expression of STAT1 phosphorylation was upregulated. Our vitro experiments revealed a significant upregulation of STAT1 phosphorylation following lactate treatment, indicating that the acetylation of STAT1 is primarily mediated by LPCAT2 rather than HDAC9 in the presence of lactate. Therefore, the results we obtained above supported that AARS1 and HDAC9 are the lactyltransferase and delactylase of LPCAT2.

This study explored PDK4-mediated lactate regulation in SI-ALI and LPCAT2 lactylation-driven ferroptosis. However, several limitations do exist: (1) unclear mechanism of exogenous lactate accumulation in SI-ALI; (2) lack of in-depth exploration of AARS1/HDAC9 interaction with LPCAT2; (3) absence of clinical safety evaluation of PDK4-in. Further research is needed to address these gaps and assess clinical feasibility.

## Conclusions

In summary, this study underscores the role of lactate in inducing damage to epithelial cells during SI-ALI. It also reveals the efficacy of the PDK4-in in curtailing lactate levels in SI-ALI. Additionally, we have elucidated the mechanism by which LPCAT2 lactylation modulates the STAT1/SLC7A11 axis, ultimately leading to ferroptosis. Collectively, our findings suggest that targeting PDK4 and LPCAT2 lactylation could potentially serve as a promising therapeutic strategy for mitigating lung injury in septic patients.

## Supplementary information

Supplementary material

Original western blots

Proteomic analysis data

## Data Availability

The data that support the findings of this study are available from the corresponding author upon reasonable request.
