# Supplementary material for: PDK4-driven lactate accumulation facilitates LPCAT2 lactylation to exacerbate sepsis-induced acute lung injury
Source: Cell Death Differ. 2025 Oct 7;33(3):557–73. doi: 10.1038/s41418-025-01585-6 (PMC13035903; doi:10.1038/s41418-025-01585-6)
Supplement: Supplementary file 1 — Supplementary material [file 41418_2025_1585_MOESM1_ESM.pdf]

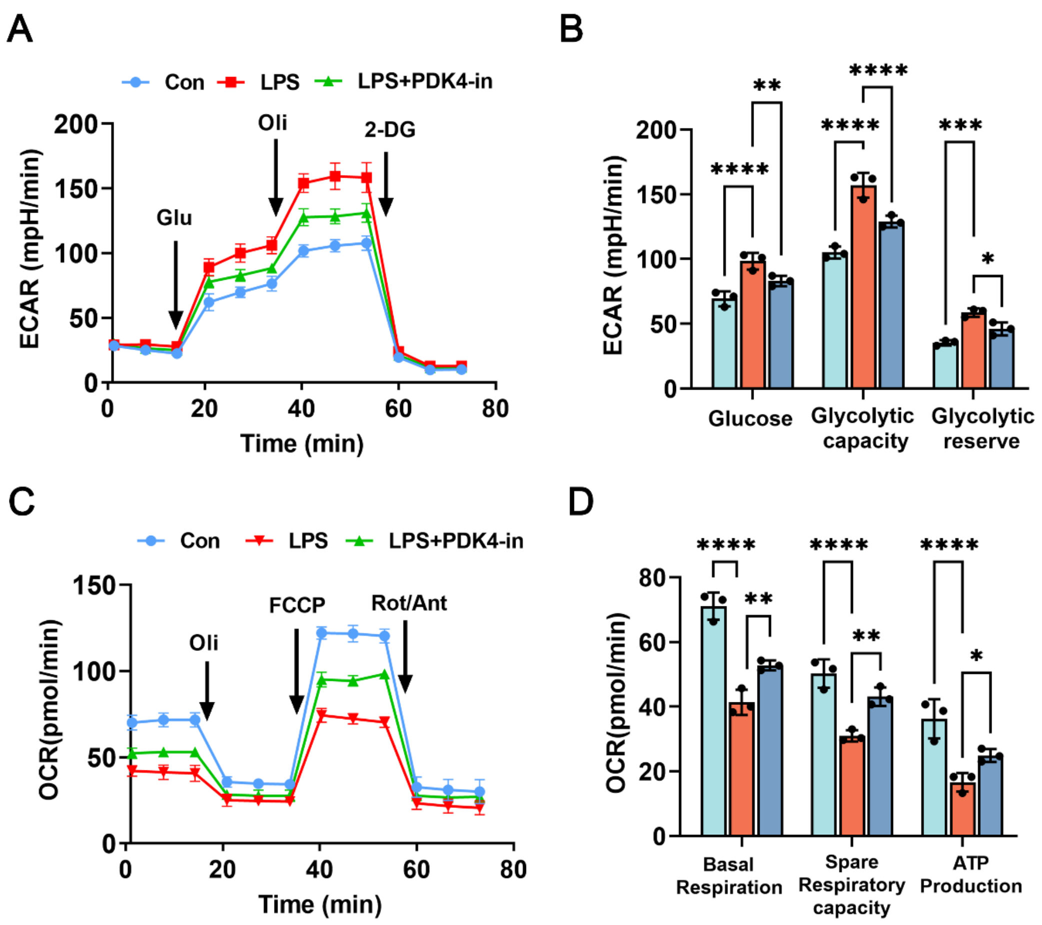

Fig S1. PDK4 can mediate glycolytic metabolic reprogramming. (A) The ECAR curve. (n=3). (B) The glycolysis, glycolytic capacity, and glycolytic reserve in the OCR curve. (C) The OCR curve. (n=3). (D) The basal respiration, spare respiratory capacity, and ATP production in the ECAR curve. Data are presented as mean ± SD. *p <0.05, **p <0.01, ***p <0.001, and ****p <0.0001. Statistical significance was determined by two-way ANOVA. Each experiment was conducted with three independent biological replicates.

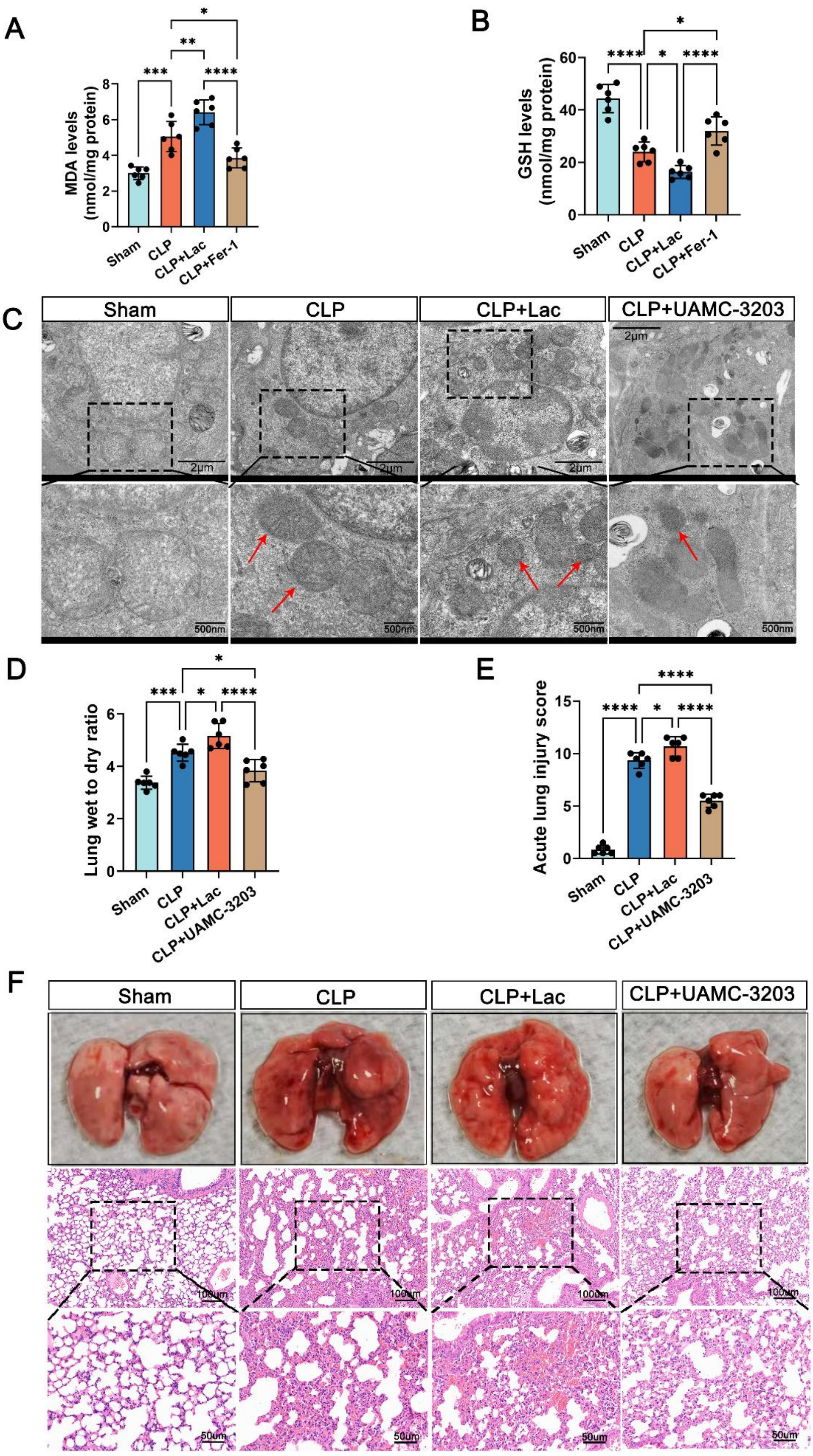
A
MDA levels
(nmol/mg protein)
Sham
CLP
CLP+Lac
CLP+Fer-1
B
GSH levels
(nmol/mg protein)
Sham
CLP
CLP+Lac
CLP+Fer-1
C
Sham
CLP
CLP+Lac
CLP+UAMC-3203
2μm
500nm
D
Lung wet to dry ratio
Sham
CLP
CLP+Lac
CLP+UAMC-3203
E
Acute lung injury score
Sham
CLP
CLP+Lac
CLP+UAMC-3203
F
Sham
CLP
CLP+Lac
CLP+UAMC-3203
100um
50um

Fig S2. Lactate exacerbates SI-ALI and UAMC-3203 mitigates SI-ALI. (A-B) The levels of MDA and GSH in the sham and CLP groups with or without lactate/UAMC-3203 administration. (n=6). (C) The representative images of TEM showed mitochondrial changes in lung tissues in the sham and CLP groups with or without lactate/ UAMC-3203 administration. (n=3, up: scale bar =2 μm; down: scale bar =500 nm). (D) Wet-to-dry ratio of lungs in the sham and CLP group with or without lactate/ UAMC-3203 administration. (n =6). (E-F) Acute lung injury score in the sham and CLP group with or without lactate/ UAMC-3203 administration. (n=6, up: scale bar =100 μm; down: scale bar =50 μm). Data are presented as mean ± SD. *p <0.05, **p <0.01, ***p <0.001, and ****p <0.0001. Statistical significance was determined by one-way ANOVA. Each experiment was conducted with six independent biological replicates.

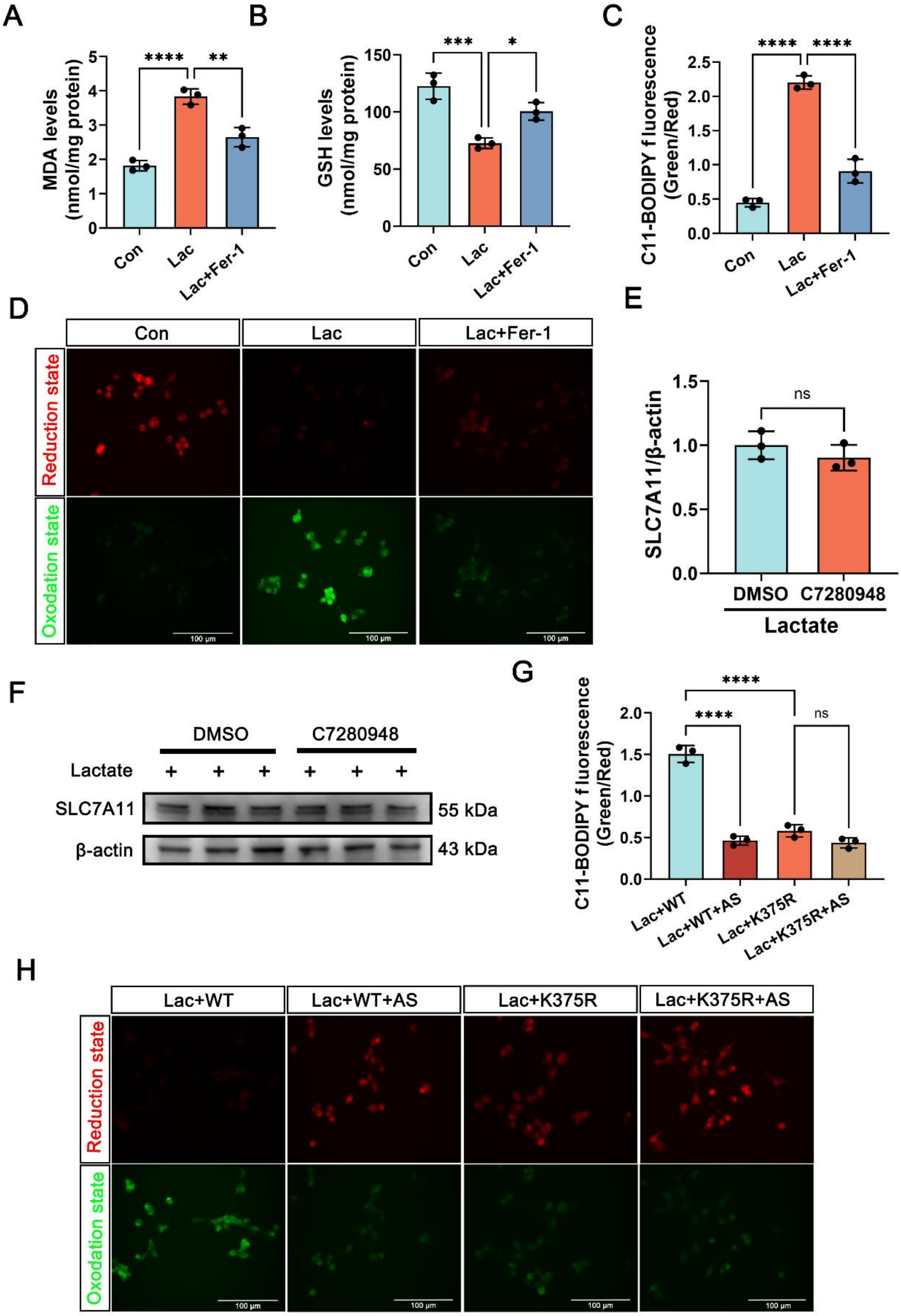

A
MDA levels (nmol/mg protein)
****
**
Con
Lac
Lac+Fer-1
B
GSH levels (nmol/mg protein)
***
*
Con
Lac
Lac+Fer-1
C
C11-BODIPY fluorescence (Green/Red)
****
****
Con
Lac
Lac+Fer-1
D
Con
Lac
Lac+Fer-1
Reduction state
Oxodation state
100 μm
E
SLC7A11/β-actin
ns
DMSO
C7280948
Lactate
F
DMSO
C7280948
Lactate
SLC7A11
55 kDa
β-actin
43 kDa
G
C11-BODIPY fluorescence (Green/Red)
****
****
ns
Lac+WT
Lac+WT+AS
Lac+K375R
Lac+K375R+AS
H
Lac+WT
Lac+WT+AS
Lac+K375R
Lac+K375R+AS
Reduction state
Oxodation state
100 μm

Fig S3. Fer-1 effectively mitigates SI-ALI, whereas the LPCAT2-STAT1-SLC7A11 axis is likely the primary driver of lipid peroxidation in LPCAT2-regulated pathways under high lactate. (A-B) The levels of MDA and GSH in the Con and Lac groups with or without Fer-1 treatment in MLE12 cells. (n=3). (C-D) Representative images and quantitative analysis of C11-BODIPY 581/591 staining in Con and Lac groups with or without lactate treatment in MLE12 cells. (n=3, scale bar =100 μm). (E-F) The expression of SLC7A11 in MLE12 cells with or without C7280948 treatment under high lactate conditions. (n=3). (G-H) Representative images and quantitative analysis of C11-BODIPY 581/591 staining in the WT and K375R group with or without AS treatment in BEAS-2B cells under high lactate conditions. (n=3, scale bar =100 μm). Data are presented as mean ± SD. *p <0.05, **p <0.01, ***p <0.001, and ****p <0.0001. Statistical significance was determined by one-way ANOVA or Student's t-test as appropriate. Each experiment was conducted with three independent biological replicates.

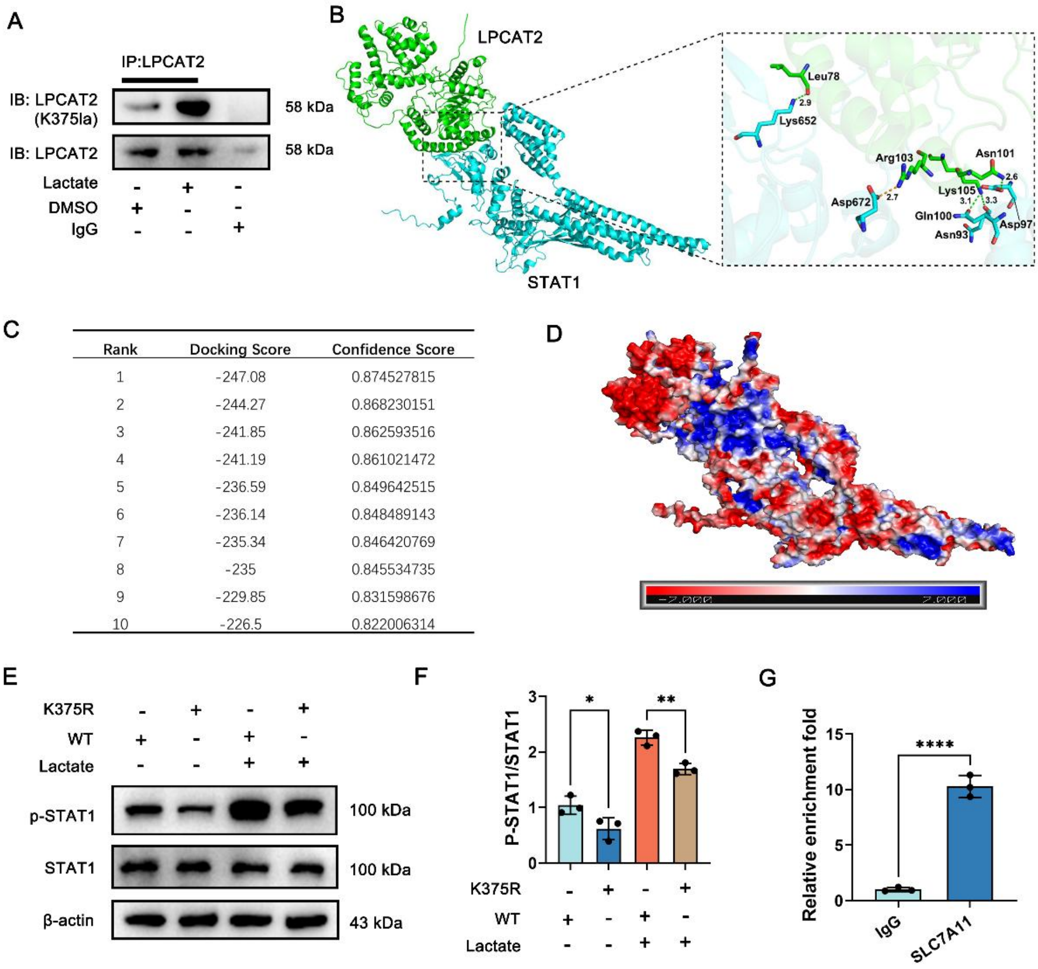

Fig S4. LPCAT2-K375 suppresses SLC7A11 expression by promoting STAT1 phosphorylation. (A) Co-IP result showed the interaction of LPCAT2-K375 with LPCAT2 in BEAS-2B cells with or without lactate treatment. (n=3). (B) The HDOCK software was utilized to predict the 3D structural model of the LPCAT2/STAT1 interaction. (C)The docking score and confidence score for the LPCAT2/STAT1 interaction were predicted using the HDOCK software. (D) The Adaptive Poisson-Boltzmann Solver was employed to analyze the electrostatic properties of the LPCAT2/STAT1 interaction. (E-F) The expression of p-STAT1 and STAT1 in the WT and K375R group with or without lactate treatment in BEAS-2B cells. (n=3). (G) CHIP

assay demonstrated a transcriptional regulatory interaction between STAT1 and the SLC7A11 promoter. Data are presented as mean ± SD. *p <0.05, and **p <0.01. Statistical significance was determined by one-way ANOVA. Each experiment was conducted with three independent biological replicates.

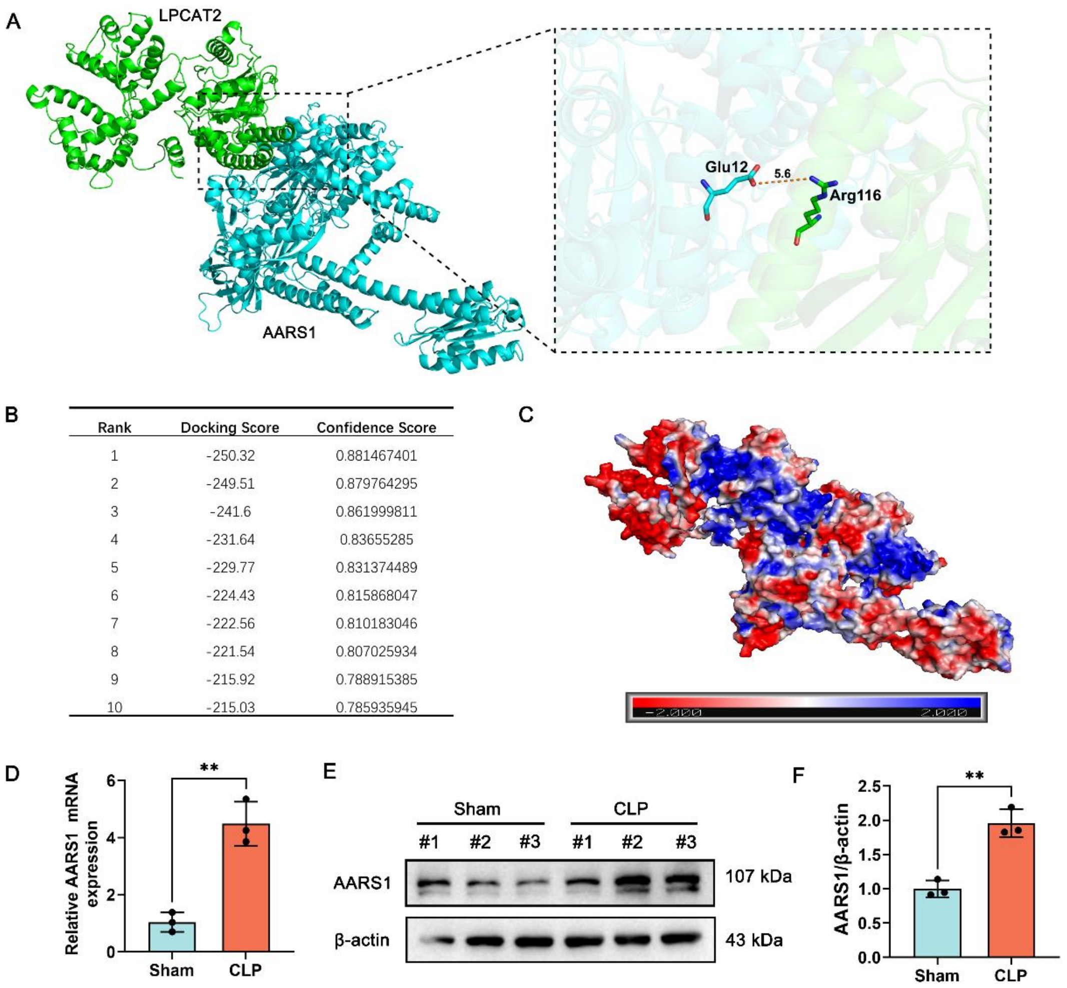

Fig S5. AARS1 is the lactyltransferase of LPCAT2-K375. (A) The HDOCK software was utilized to predict the 3D structural model of the AARS1/LPCAT2 interaction. (B) The docking score and confidence score for the LPCAT2/STAT1 interaction were predicted using the HDOCK software. (C) The Adaptive Poisson-Boltzmann Solver

was employed to analyze the electrostatic properties of the AARS1/LPCAT2 interaction. (D) The levels of AARS1 mRNA in lung tissues in the sham and CLP groups. (n=3). (E-F) The expression of AARS1 in the sham and CLP groups. (n=3). Data are presented as mean ± SD. **p <0.01. Statistical significance was determined by Student's t-test as appropriate. Each experiment was conducted with three independent biological replicates.

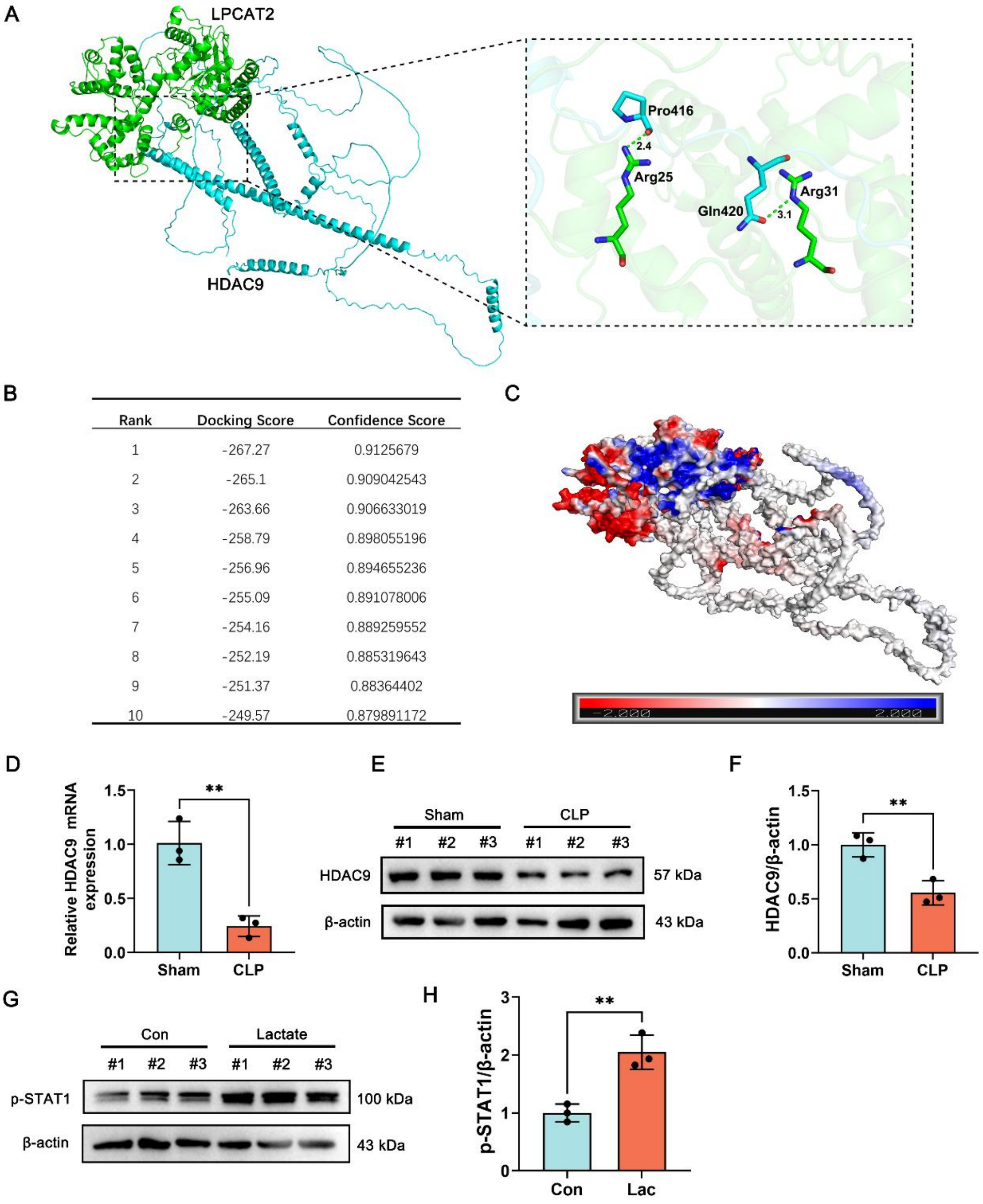

Rank	Docking Score	Confidence Score
1	-267.27	0.9125679
2	-265.1	0.909042543
3	-263.66	0.906633019
4	-258.79	0.898055196
5	-256.96	0.894655236
6	-255.09	0.891078006
7	-254.16	0.889259552
8	-252.19	0.885319643
9	-251.37	0.88364402
10	-249.57	0.879891172

Fig S6. HDAC9 is the delactylase of LPCAT2-K375. (A) The HDOCK software was utilized to predict the 3D structural model of the HDAC9/LPCAT2 interaction. (B) The docking score and confidence score for the HDAC9/LPCAT2 interaction were predicted using the HDOCK software. (C) The Adaptive Poisson-Boltzmann Solver was employed to analyze the electrostatic properties of the HDAC9/LPCAT2 interaction. (D) The levels of HDAC9 mRNA in lung tissues in the sham and CLP groups. (n=3). (E-F) The expression of AARS1 in the sham and CLP groups. (n=3). (G-H) The expression levels of p-STAT1 with or without lactate treatment in BEAS-2B cells. (n=3). Data are presented as mean ± SD. **p <0.01. Statistical significance was determined by Student's t-test as appropriate. Each experiment was conducted with three independent biological replicates.

Supplementary file 1：Supplementary methods for lactylation proteomics and proteomic Analysis

We employed cutoff values of fold change (FC) > 1.5 and p-value < 0.05 to identify differentially expressed proteins. To achieve an in-depth understanding of the functional characteristics of various proteins, we conducted a comprehensive functional annotation analysis on the identified proteins. This analysis covered several key aspects, including gene ontology (GO) annotation, Kyoto Encyclopedia of Genes and Genomes (KEGG) analyses and subcellular localization of the proteins.

For protein quantitative analysis, the relative quantification value of each protein is calculated based on the signal intensity values provided in the search results for different samples. Firstly, the original signal intensity of the protein in each sample is transformed using a Log2 logarithmic transformation, with the formula *I*ij=log2 (Intensity ij), where *I* represents the sample and *J* represents the protein. Then, a centralization transformation is performed on the log2-transformed values (*I*) for each protein to obtain the relative quantification value (*U*), calculated as *U* ij=*I* ij−Mean (Lj). Finally, to eliminate systematic errors in the sample size of different samples in mass spectrometry detection, the relative quantification value (*U*) is corrected using the median normalization method (NR), with the formula *NR* ij=*U* ij-Median (Ui).

The color scale employed in these heatmaps encompasses: #E41A1C, #377EB8, #4DAF4A, #984EA3, #FF7F00, #FFFF33, #A65628, #F781BF.

Supplementary Table 1: Details of qPCR primers.

Gene	Forward (5’-3’)	Reverse (3’-5’)
PDK4	CCGCTGTCCATGAAGCA	GCAGAAAAGCAAAGGACGTT
SLC7A11	CCTCTGCCAGCTGTTATTGTT	CCTGGCAAAACTGAGGAAAT
AARS1	TCACCCAAGAGTTTGGCATTC	CCTGGGAGGATTTTGGTGTCA
HDAC9	AAGAAGCGAGTGTTTGAGGTG	GTTTGGTGAACTGGGACCTG
β-actin	GGCTGTATTCCCCTCCATCG	CCAGTTGGTAACAATGCCATGT

Supplementary Table 2: Details of Antibodies.

Antibodies name	Source	Identifier
Anti - L- Lactyl Lysine	PTMBIO	Cat# PTM-1401
Anti-Acetyllysine	PTMBIO	Cat# PTM-101
Anti-LPCAT2	Santa Cruz	Cat# sc-514354
Anti-LPCAT2-K375	Keya Biotech	Cat# PABDZ005
Anti-PDK4	Proteintech	Cat# 12949-1-AP
Anti-SLC7A11	Immunoway	Cat# 26864-1-AP
Anti-β-tubulin	Ray Antibody Biotech	Cat# RM2003
Anti-β-actin	Proteintech	Cat# 20536-1-AP
Anti-HDAC9	Proteintech	Cat# 28334-1-AP
Anti-AARS1	Proteintech	Cat# 17394-1-AP
Anti-STAT1	Cell Signaling Technology	Cat# 14994
Anti-p-STAT1	Cell Signaling Technology	Cat# 9167
Anti-Flag	Immunoway	Cat# YM3809

Supplementary Table 3: The list of proteins under the functional enrichments

**Functional enrichments**	**Proteins**
Lipid oxidation	Abcd3; Prkaa1; Hadha; Acaa2; Acat1; Hadhb; Cygb; Echdc1; Acadsb; Acoxl; Acox3; Eci2
NADPH regeneration	Pfkm; Pkm
Ferroptosis	GPX4
