# Supplementary material for: PDK4-driven lactate accumulation facilitates LPCAT2 lactylation to exacerbate sepsis-induced acute lung injury
Source: Cell Death Differ. 2025 Oct 7;33(3):557–73. doi: 10.1038/s41418-025-01585-6 (PMC13035903; doi:10.1038/s41418-025-01585-6)
Supplement: Supplementary file 2 — Original western blots [file 41418_2025_1585_MOESM2_ESM.pdf]

Fig. 2

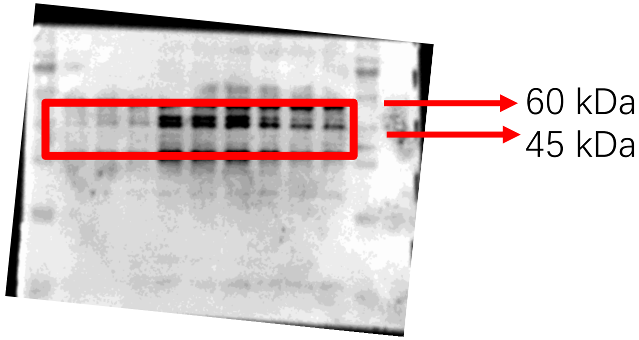

Fig 2H：PDK4

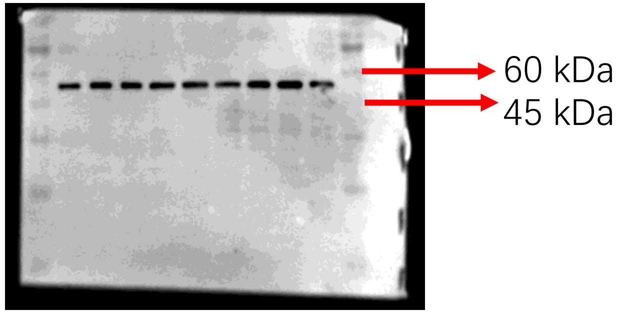

Fig 2H：Tubulin

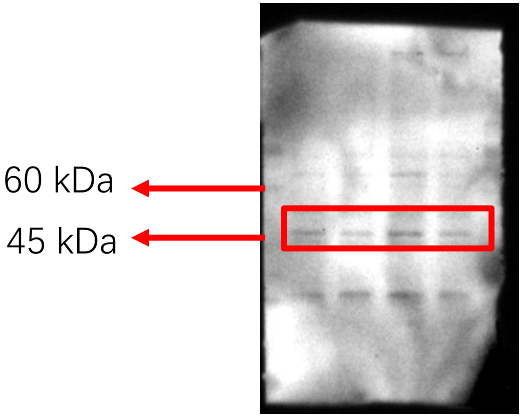

Fig 2R：PDK4

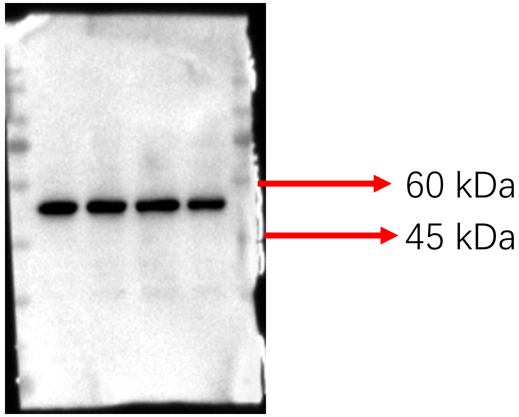

Fig 2R：Tubulin

Fig. 4

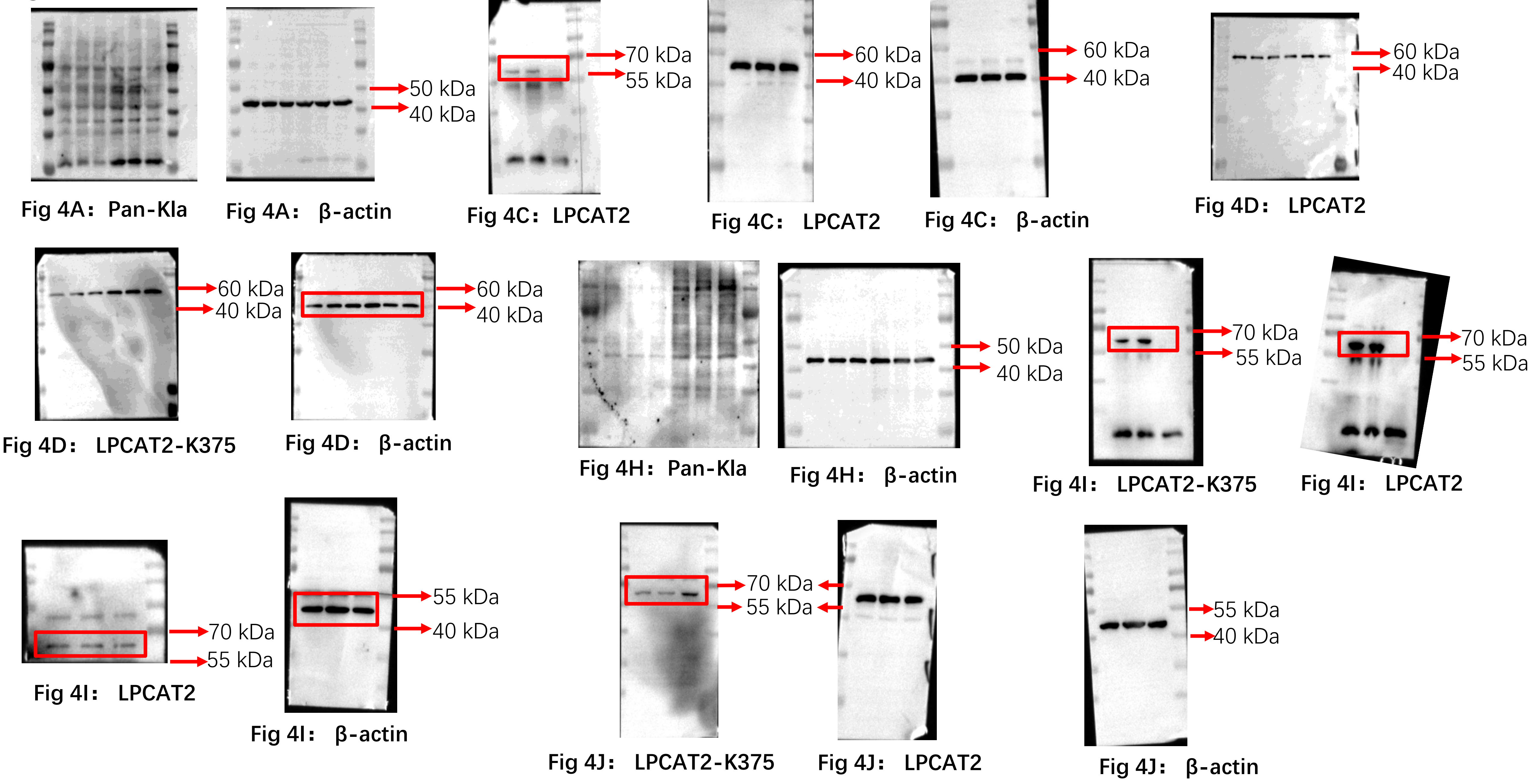

Fig 4A：Pan-Kla
50 kDa
40 kDa
Fig 4A： β-actin
70 kDa
55 kDa
Fig 4C：LPCAT2
60 kDa
40 kDa
Fig 4C： LPCAT2
60 kDa
40 kDa
Fig 4C： β-actin
60 kDa
40 kDa
Fig 4D： LPCAT2
60 kDa
40 kDa
Fig 4D： LPCAT2-K375
60 kDa
40 kDa
Fig 4D： β-actin
Fig 4H：Pan-Kla
50 kDa
40 kDa
Fig 4H： β-actin
70 kDa
55 kDa
Fig 4I： LPCAT2-K375
70 kDa
55 kDa
Fig 4I： LPCAT2
70 kDa
55 kDa
Fig 4I： LPCAT2
55 kDa
40 kDa
Fig 4I： β-actin
70 kDa
55 kDa
Fig 4J： LPCAT2-K375
Fig 4J： LPCAT2
55 kDa
40 kDa
Fig 4J： β-actin

Fig. 5

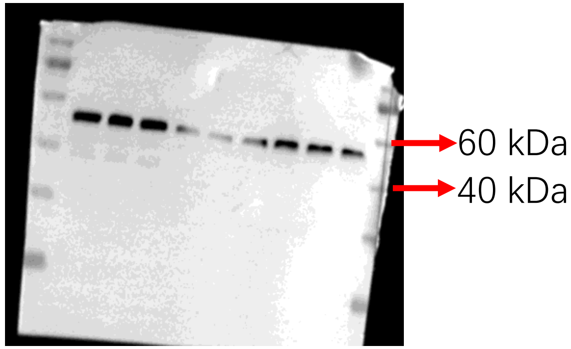

Fig 5B：SLC7A11

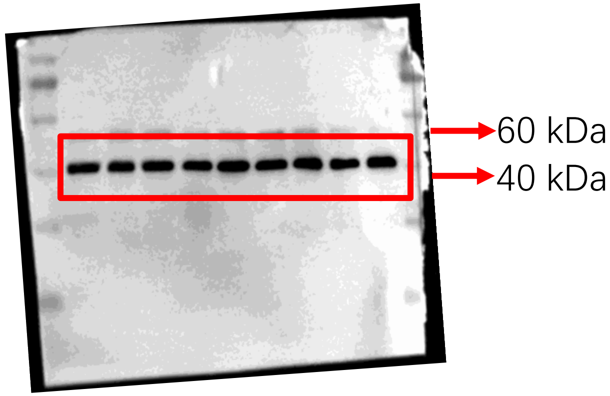

Fig 5B：β-actin

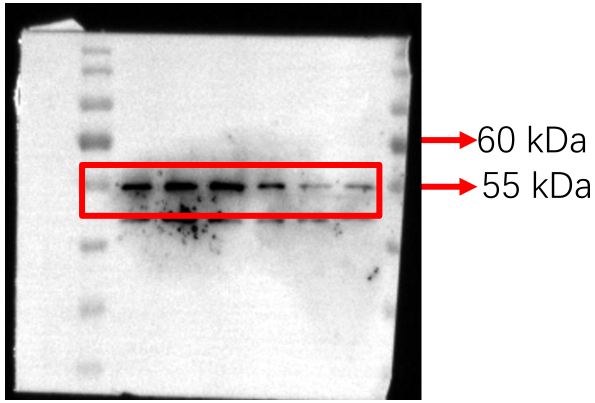

Fig 5G：SLC7A11

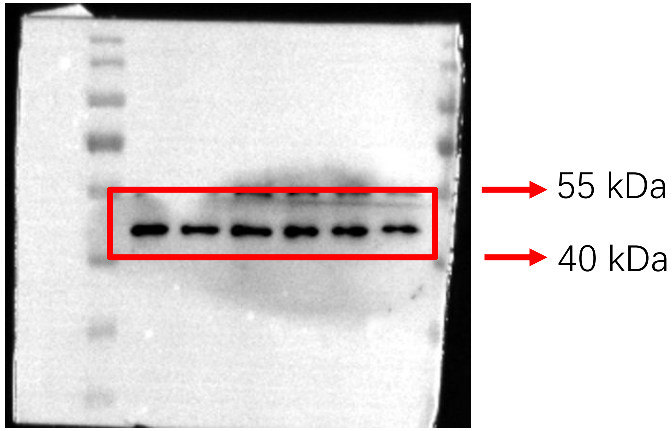

Fig 5G：β-actin

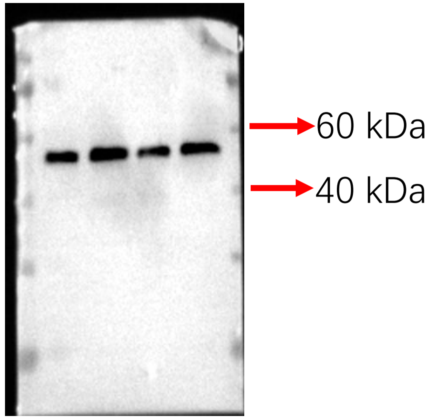

Fig 5I：SLC7A11

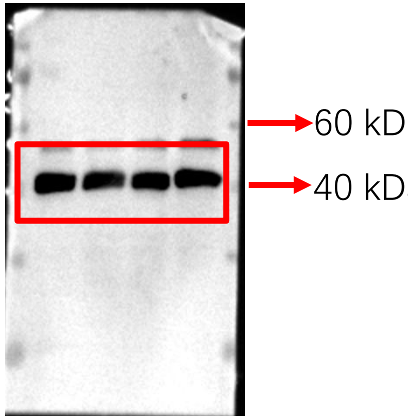

Fig 5I：β-actin

Fig. 6

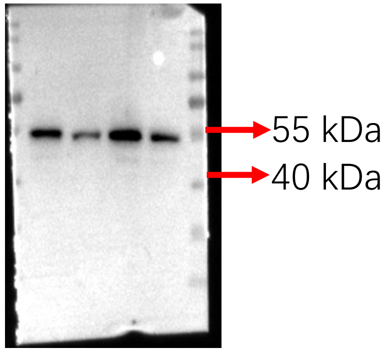

Fig 6A：LPCAT2-K375

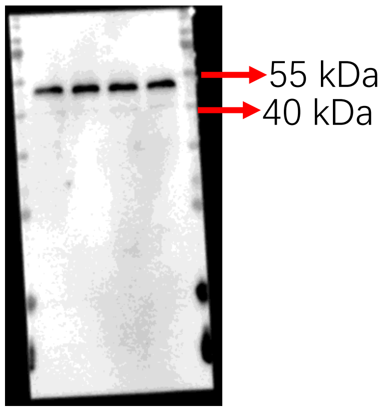

Fig 6A：LPCAT2

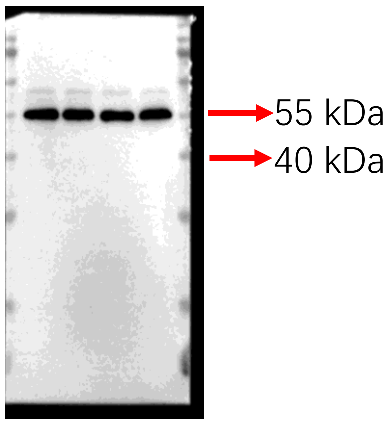

Fig 6A：β-actin

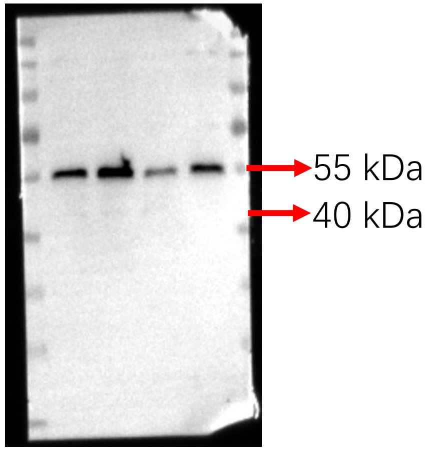

Fig 6H：SLC7A11

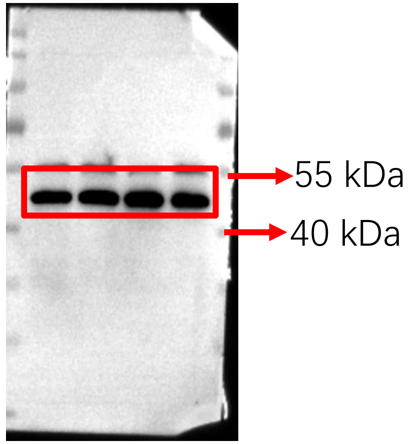

Fig 6H：β-actin

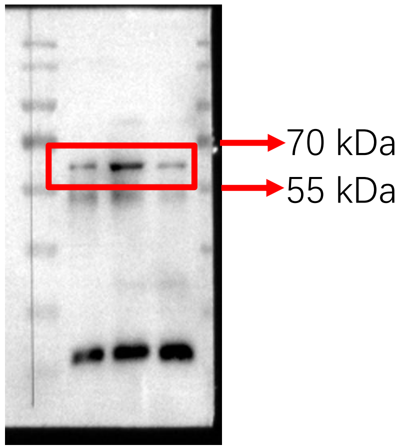

Fig 6C：LPCAT2-K375

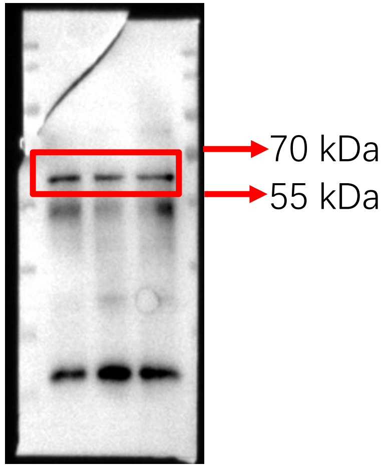

Fig 6C：LPCAT2

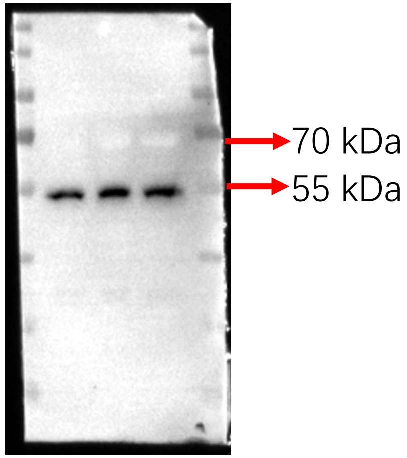

Fig 6C：LPCAT2（input）

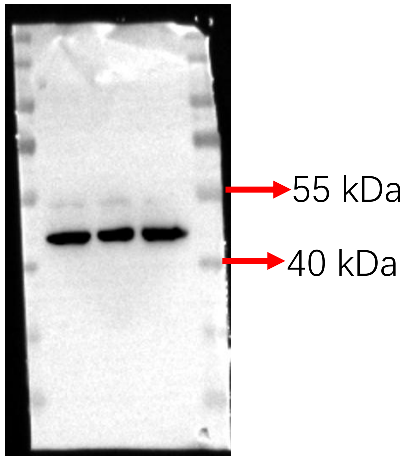

Fig 6C：β-actin

Fig. 7

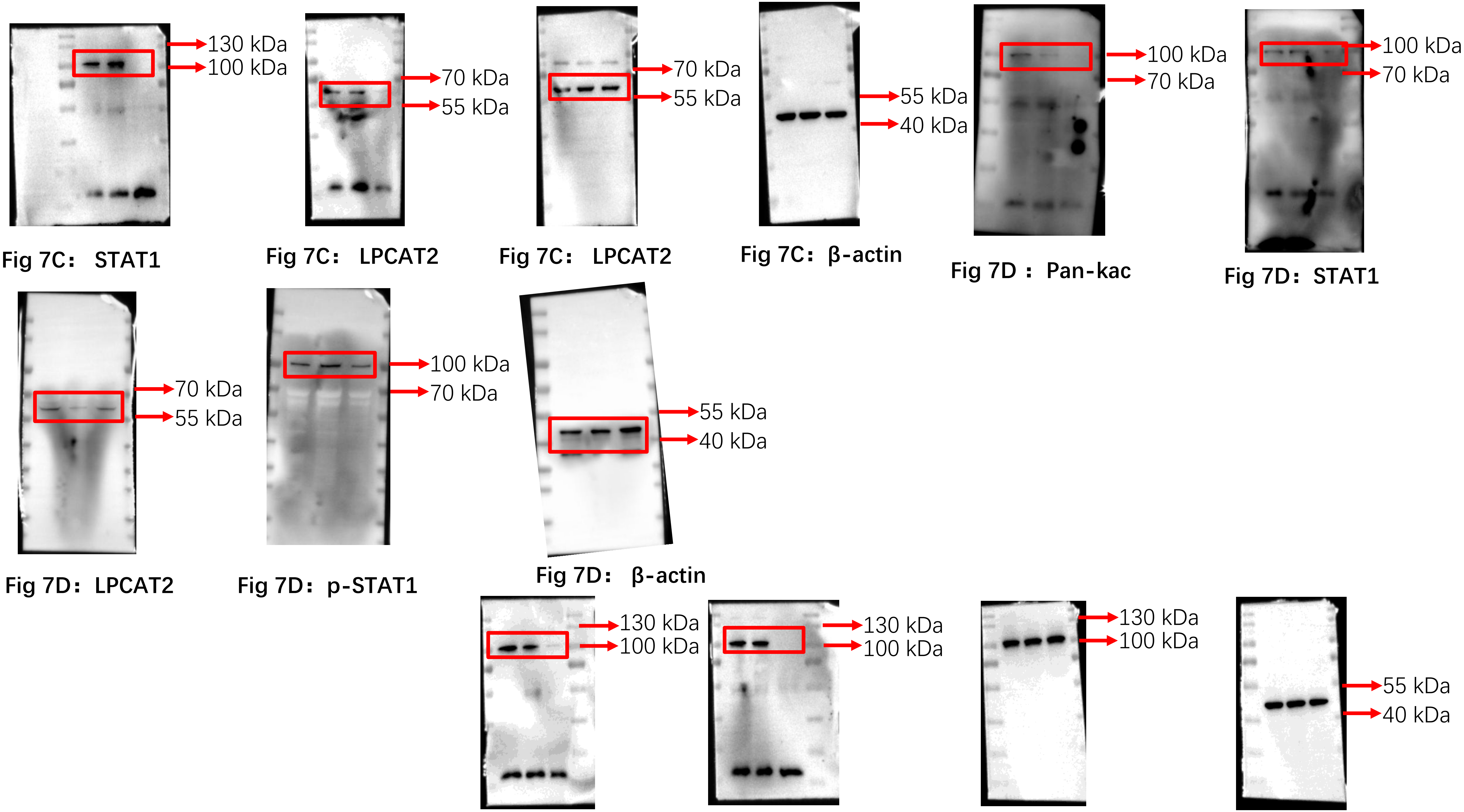

Fig 7C: STAT1

Fig 7C: LPCAT2

Fig 7C: LPCAT2

Fig 7C: β-actin

Fig 7D : Pan-kac

Fig 7D: STAT1

Fig 7D: LPCAT2

Fig 7D: p-STAT1

Fig 7D: β-actin

Fig 7E: Pan-Kac

Fig 7E: STAT1

Fig 7E: STAT1

Fig 7E: β-actin

Fig. 8

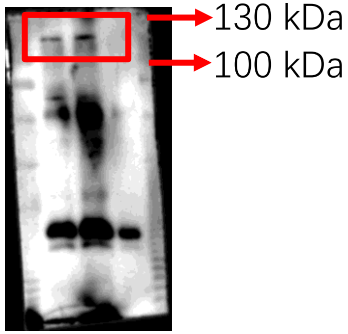

Fig 8B： AARS1

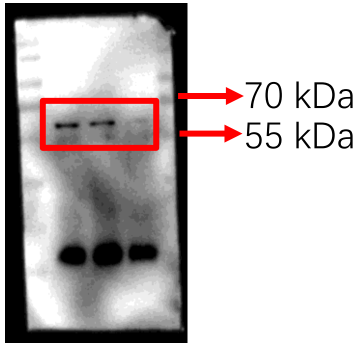

Fig 8B： LPCAT2

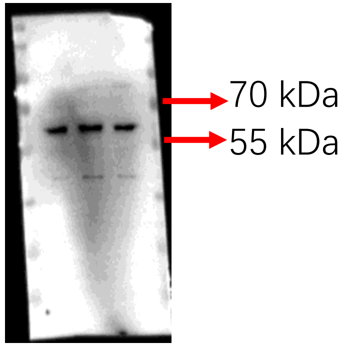

Fig 8B： LPCAT2

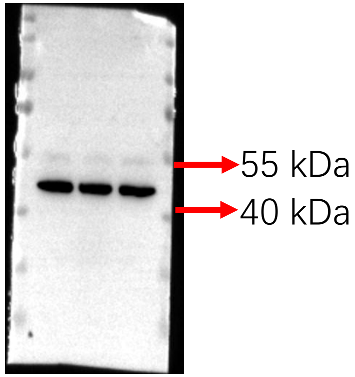

Fig 8B：β-actin

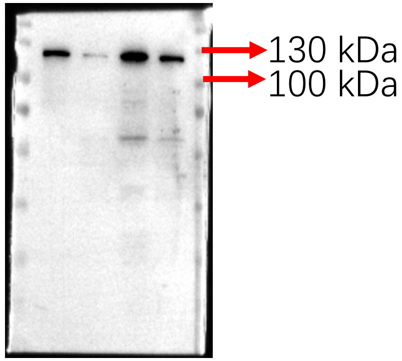

Fig 8C： AARS1

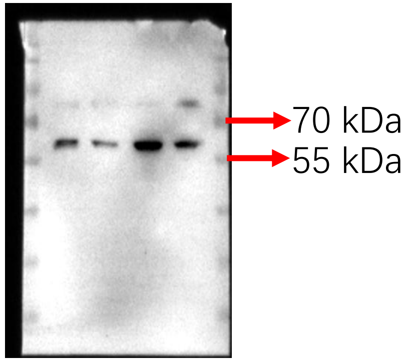

Fig 8C： LPCAT2-K375

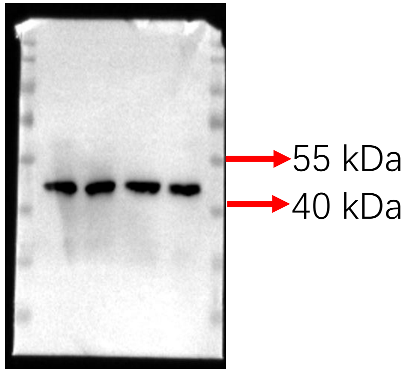

Fig 8C：β-actin

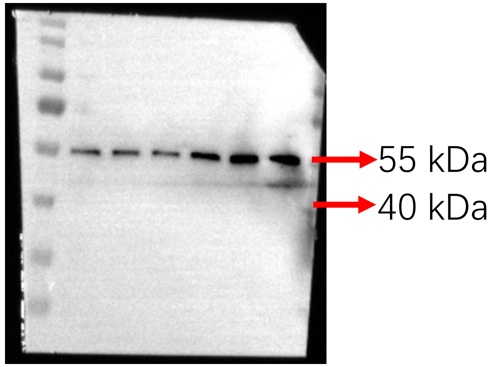

Fig 8G： SLC7A11

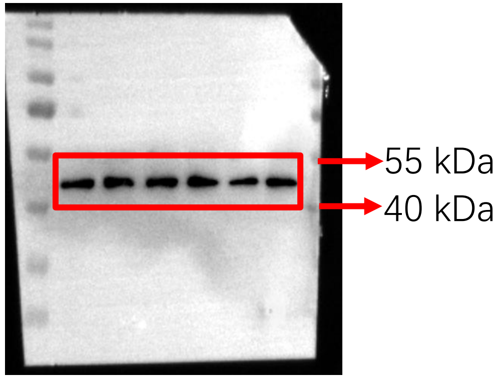

Fig 8G： β-actin

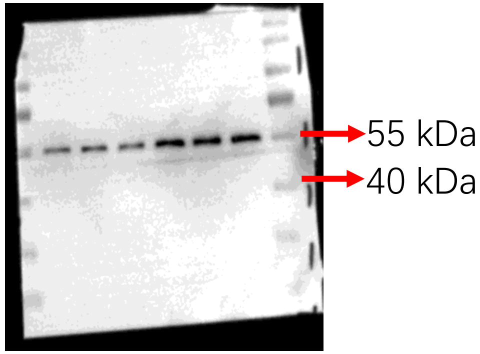

Fig 8J： SLC7A11

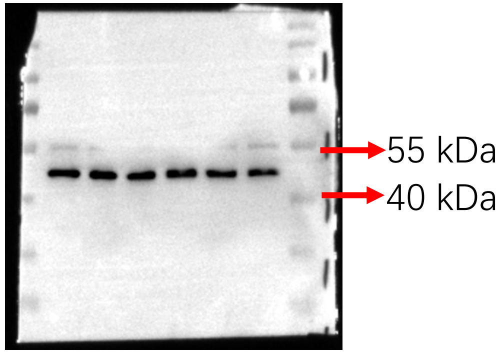

Fig 8J： β-actin

Fig. 8

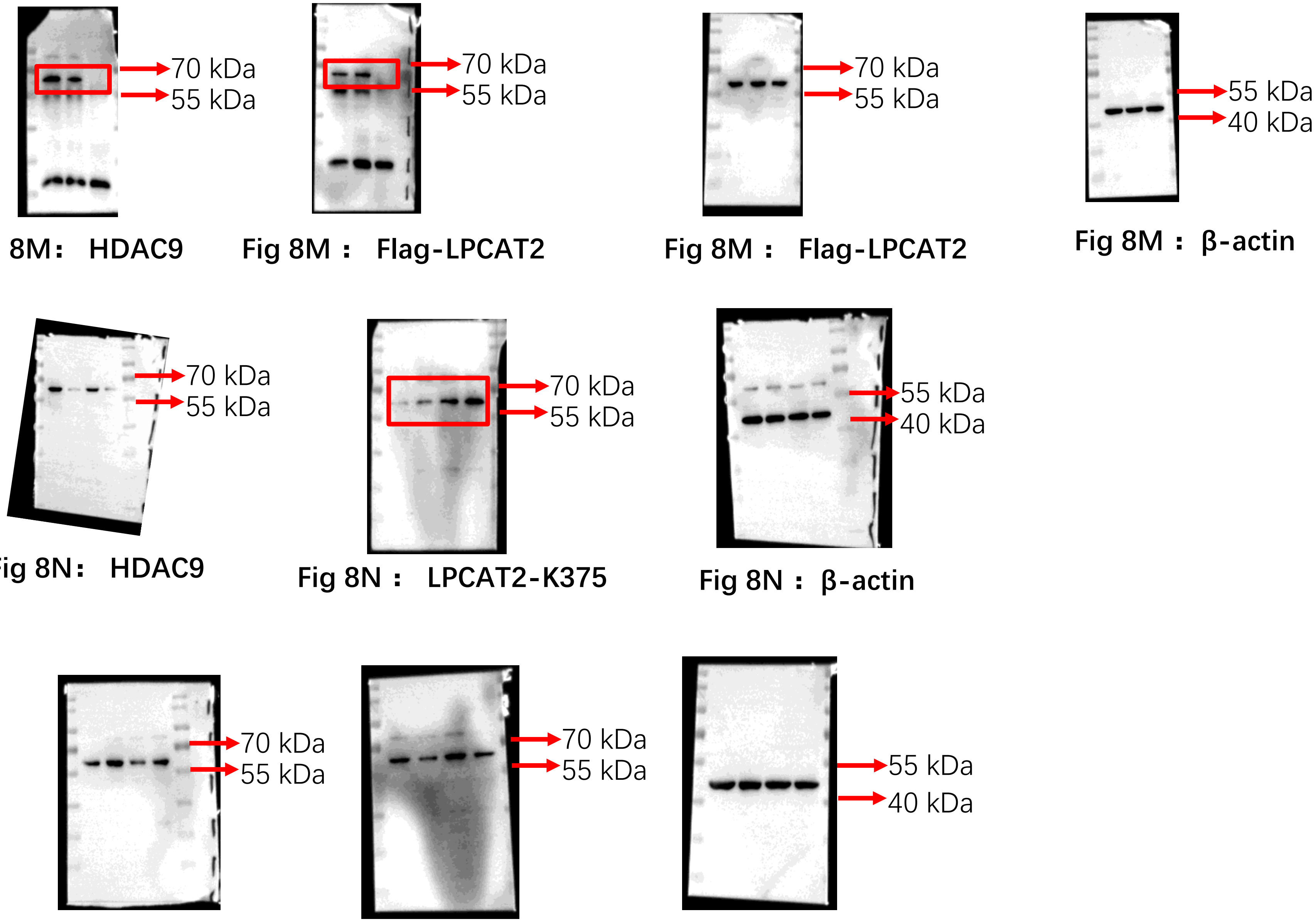

Fig 8M： HDAC9
Fig 8M ： Flag-LPCAT2
Fig 8M ： Flag-LPCAT2
Fig 8M ： β-actin
Fig 8N： HDAC9
Fig 8N ： LPCAT2-K375
Fig 8N ： β-actin
Fig 8Q： HDAC9
Fig 8Q ： LPCAT2-K375
Fig 8Q： β-actin

Fig. S3

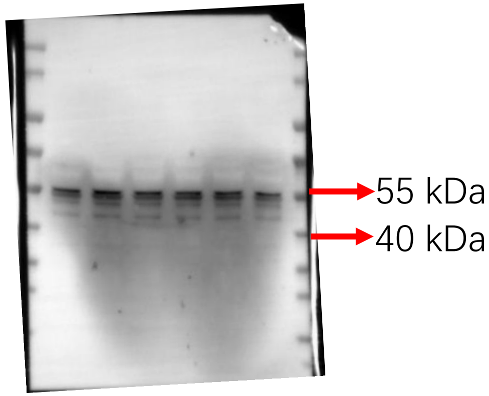

Fig S3F: SLC7A11

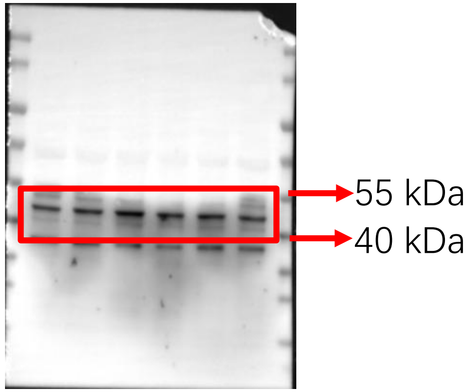

Fig S3F: β-actin

Fig. S4

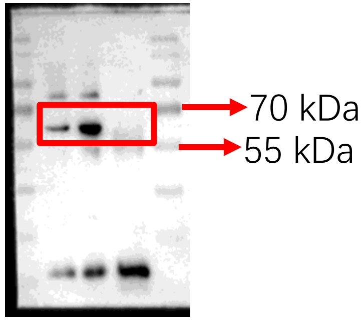

Fig S4A: LPCAT2-K375

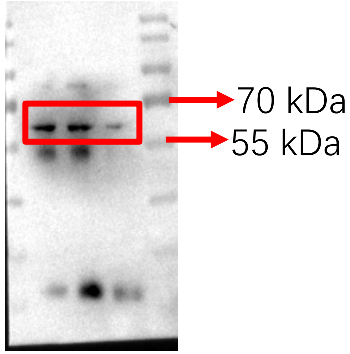

Fig S4A: LPCAT2

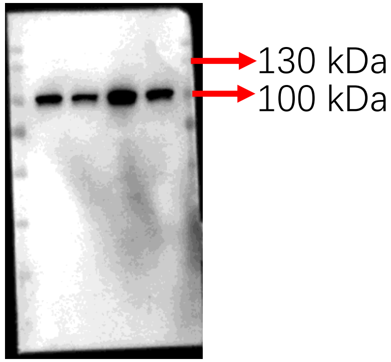

Fig S4E: p-STAT1

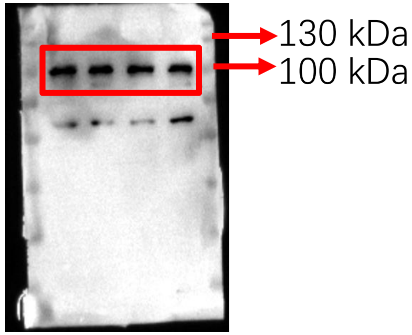

Fig S4E: STAT1

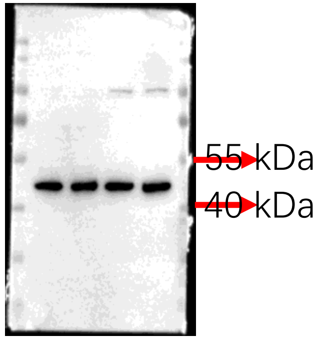

Fig S4E: β-actin

Fig. S5

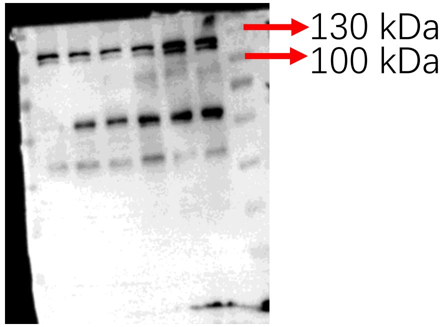

Fig S5E： AARS1

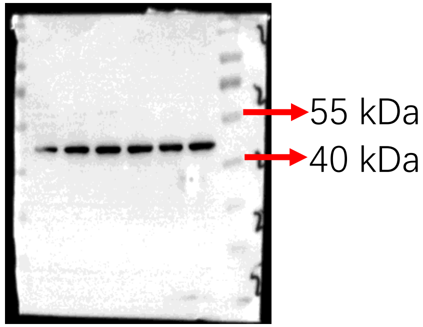

Fig S5E： β-actin

Fig. S6

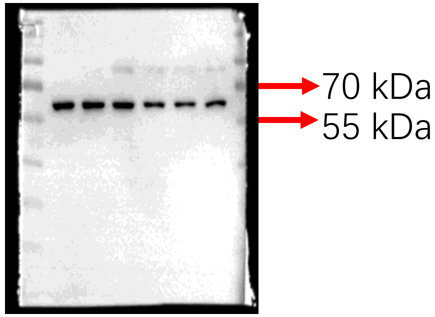

Fig S6E： HDAC9

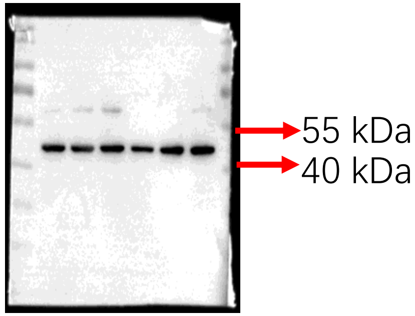

Fig S6E： β-actin

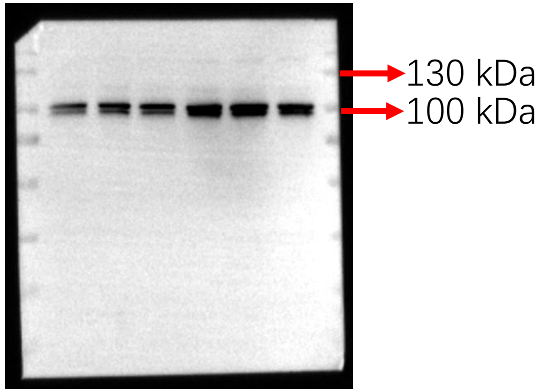

Fig S6G： p-STAT1

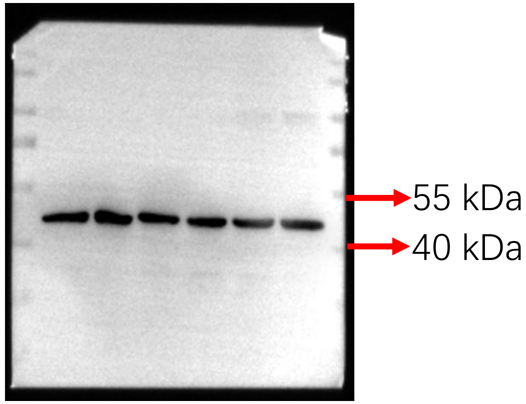

Fig S6G： β-actin
